# The TNFR superfamily member Fn14 impacts immunity and survival in experimental gliomas and response to immune checkpoint inhibitor therapy in glioblastoma patients

**DOI:** 10.1093/noajnl/vdag023

**Published:** 2026-02-12

**Authors:** Pranjali P Kanvinde, Adarsha P Malla, Alexandra A Seas, Emylee McFarland, Jennifer R Fang, Matthew J Flick, Nina P Connolly, Angad Beniwal, Nima Sharifai, Chixiang Chen, Manuel Yepes, Eli E Bar, Pavlos Anastasiadis, Nhan L Tran, Jeffrey A Winkles, Graeme F Woodworth

**Affiliations:** Department of Neurosurgery, University of Maryland School of Medicine, Baltimore, Maryland, USA; Brain Tumor Program, University of Maryland Marlene and Stewart Greenebaum Cancer Center, Baltimore, Maryland, USA; Department of Neurosurgery, University of Maryland School of Medicine, Baltimore, Maryland, USA; Brain Tumor Program, University of Maryland Marlene and Stewart Greenebaum Cancer Center, Baltimore, Maryland, USA; Department of Neurosurgery, University of Maryland School of Medicine, Baltimore, Maryland, USA; Brain Tumor Program, University of Maryland Marlene and Stewart Greenebaum Cancer Center, Baltimore, Maryland, USA; Department of Neurosurgery, University of Maryland School of Medicine, Baltimore, Maryland, USA; Brain Tumor Program, University of Maryland Marlene and Stewart Greenebaum Cancer Center, Baltimore, Maryland, USA; Department of Neurosurgery, University of Maryland School of Medicine, Baltimore, Maryland, USA; Brain Tumor Program, University of Maryland Marlene and Stewart Greenebaum Cancer Center, Baltimore, Maryland, USA; Department of Cancer Biology, Mayo Clinic Arizona, Phoenix, Arizona, USA; Mayo Clinic Alix School of Medicine, Mayo Clinic Arizona, Phoenix, Arizona, USA; Department of Radiology, Mayo Clinic Arizona, Phoenix, Arizona, USA; Department of Neurosurgery, University of Maryland School of Medicine, Baltimore, Maryland, USA; Brain Tumor Program, University of Maryland Marlene and Stewart Greenebaum Cancer Center, Baltimore, Maryland, USA; Mayo Clinic Alix School of Medicine, Mayo Clinic Arizona, Phoenix, Arizona, USA; Department of Pathology, University of Maryland School of Medicine, Baltimore, Maryland, USA; Department of Neurosurgery, University of Maryland School of Medicine, Baltimore, Maryland, USA; Department of Epidemiology & Public Health, University of Maryland School of Medicine, Baltimore, Maryland, USA; Division of Neuropharmacology and Neurologic Diseases, Emory Primate Research Center, Atlanta, Georgia, USA; Department of Neurology & Center for Neurodegenerative Disease, Emory University, Atlanta, Georgia, USA; Department of Neurology, Veterans Affairs Medical Center, Atlanta, Georgia, USA; Department of Neurosurgery, University of Maryland School of Medicine, Baltimore, Maryland, USA; Brain Tumor Program, University of Maryland Marlene and Stewart Greenebaum Cancer Center, Baltimore, Maryland, USA; Department of Neurosurgery, University of Maryland School of Medicine, Baltimore, Maryland, USA; Brain Tumor Program, University of Maryland Marlene and Stewart Greenebaum Cancer Center, Baltimore, Maryland, USA; Department of Diagnostic Radiology and Nuclear Medicine, University of Maryland School of Medicine, Baltimore, Maryland, USA; Fischell Department of Bioengineering, University of Maryland, College Park, Maryland, USA; Department of Cancer Biology, Mayo Clinic Arizona, Phoenix, Arizona, USA; Department of Neurological Surgery, Mayo Clinic Arizona, Phoenix, Arizona, USA; Department of Neurosurgery, University of Maryland School of Medicine, Baltimore, Maryland, USA; Brain Tumor Program, University of Maryland Marlene and Stewart Greenebaum Cancer Center, Baltimore, Maryland, USA; Department of Neurosurgery, University of Maryland School of Medicine, Baltimore, Maryland, USA; Brain Tumor Program, University of Maryland Marlene and Stewart Greenebaum Cancer Center, Baltimore, Maryland, USA; Department of Diagnostic Radiology and Nuclear Medicine, University of Maryland School of Medicine, Baltimore, Maryland, USA; Fischell Department of Bioengineering, University of Maryland, College Park, Maryland, USA

**Keywords:** Fn14, glioblastoma, immunomodulation, immunotherapy, tumor microenvironment

## Abstract

**Background:**

Fibroblast growth factor-inducible 14 (Fn14) belongs to the TNFR superfamily. Fn14 overexpression can drive receptor-autonomous signaling, increase both cell invasion and tumor-associated macrophages/microglia (TAMMs) recruitment, and correlates with reduced survival in glioblastoma (GBM) patients and rat gliomas. While prior studies report Fn14 expression in non-tumor cells within the GBM tumor microenvironment (TME), their relative contributions to glioma pathobiology remain unclear.

**Methods:**

Using tumor-host pairings of Fn14-positive and -knockout (-KO) cells and mice, we examined the role of Fn14 in glioma biology. Mouse glioma and human GBM datasets were analyzed at the cellular, protein, and transcriptomic levels to assess Fn14-associated changes in the glioma TME and survival outcomes.

**Results:**

Fn14 was found to be highly expressed in tumor cells and TAMMs in human GBM and 2 well-characterized murine glioma models. Fn14 KO in both tumor and host cells increased overall survival. Notably, this survival benefit was greater in the glioma model characterized by a more immunologically activated TME. Immunophenotyping revealed that Fn14 loss reshapes the tumor-immune landscape, reducing the presence of immunosuppressive macrophages and exhausted T-cells, suggesting that Fn14 modulates both innate and adaptive immune responses. These findings were supported by analyses of human GBM datasets, where high Fn14 expression correlated with immunosuppressive shifts and poor patient responses to immune checkpoint inhibitor therapy.

**Conclusions:**

This study provides the first description of the contributions of both tumor- and host-derived Fn14 expression to tumor immunity and survival and identifies Fn14 as an important mediator of innate and adaptive immune responses in gliomas.

Key Points

- Both tumor and host Fn14 levels impact overall survival in glioma models.
- Fn14 KO in gliomas reduces M2-like TAMMs and exhausted T-cells.
- High Fn14 in human GBM correlates with poor response to checkpoint immunotherapy.

Importance of the Study

Glioblastoma (GBM) is the most common and lethal primary adult brain tumor and is characterized by brain invasion, immunosuppression, and treatment resistance. TWEAK/Fn14 signaling is an important driver of glioma cell survival and invasion. To date, most studies have focused on Fn14 signaling in tumor cells and its impact on glioma biology. Recent computational analyses of GBM datasets have suggested possible pro-tumorigenic and immunosuppressive contributions of Fn14-expressing stromal cells in the glioma microenvironment, but this has not been experimentally validated. This study is the first to characterize the differential and combined impact of tumor- vs host-derived Fn14 on glioma progression and survival in preclinical immunocompetent tumor models. We found that Fn14 expression impacts both innate and adaptive immunity in murine gliomas and human GBMs and alters survival. These findings indicate that depleting or inhibiting Fn14 in GBM could be a promising approach to reduce immunosuppression and improve responses to immunotherapy.

Glioblastoma (GBM) (IDH-wildtype, grade 4) is the most common and lethal primary malignant brain tumor affecting adults in the United States.^1^ Despite significant advancements in basic and translational research, the standard of care for GBM has been largely unchanged for over 2 decades, relying on maximal safe resection surgery followed by concomitant chemoradiation.^1^ Treatment failure is common, and most patients present with recurrence within a year following primary diagnosis. Thus, overall prognosis for GBM patients is dismal with a median survival of less than 18 months.^1^ The unique anatomical, pathological and molecular characteristics of GBM, namely (i) drug-impermeable blood-brain barrier, (ii) aggressively invasive tumor cells, (iii) inter- and intra-tumoral heterogeneity, and (iv) immunosuppressive tumor microenvironment (TME) pose significant challenges to successful therapy.^2^^,^^3^ Novel, biology-informed treatment approaches that can address these hurdles are sorely needed.

Fibroblast growth factor-inducible 14 (Fn14), a TNF receptor superfamily member, is a key player in driving invasive glioma biology.^4–9^ Its sole ligand is the pro-inflammatory cytokine TNF-like weak inducer of apoptosis (TWEAK).^10^ TWEAK/Fn14 signaling regulates tissue repair post-injury primarily via the NF-κB pathway, but it can also activate MAPK, PI3K/Akt, and JAK/STAT signaling.^9^^,^^10^ TWEAK/Fn14 signaling increases the expression of anti-apoptosis genes, promoting glioma cell survival and treatment resistance.^9^^,^^11^^,^^12^ Importantly, TWEAK-independent Fn14 signaling is also known and may occur once Fn14 expression levels reach certain thresholds, leading to spontaneous receptor trimerization/multimerization and uncontrolled downstream (receptor-autonomous) signaling.^9^^,^^13^^,^^14^ Fn14, minimally expressed in healthy tissues, is overexpressed in multiple human cancers, including GBMs.^9^^,^^10^ Fn14 but not TWEAK overexpression in primary and recurrent GBM patients is associated with significantly worse survival outcomes.^8^^,^^15^ Elevated Fn14 expression enhances the proliferation, migration and invasive capacity of glioma cells.^4–7^^,^^9^ Our group demonstrated that Fn14 overexpression in tumor-initiating neural precursor cells in the rat brain transforms low-grade gliomas into invasive high-grade tumors, significantly decreasing animal survival.^8^ Furthermore, elevated Fn14 expression, despite low TWEAK levels, significantly increased TAMM infiltration^8^ and non-canonical NF-κB signaling,^8^ which is known to promote glioma invasiveness.^16–19^ Importantly, since TWEAK levels have been shown to be quite low in Fn14-high rat and human GBM tumors,^4^^,^^7^^,^^8^ it is likely that the Fn14-mediated effects observed in these tumors are derived from the aforementioned ligand-independent, receptor-autonomous mechanism.

Prior studies on the TWEAK/Fn14 axis in cancer have largely focused on investigating how Fn14 signaling in tumor cells impacts disease biology. Emerging evidence from recent studies indicates that TWEAK/Fn14-mediated cellular crosstalk between tumor and stromal cells (eg, fibroblasts, macrophages and T-cells) in the TME can accelerate tumor progression,^20^ cachexia,^21^ metastasis,^22^ and immunosuppression^23–28^ in various cancers, including gliomas.^29–33^ Recent analysis of GBM patient datasets by our group revealed that Fn14 is also expressed by non-tumor cells; specifically, TAMMs, in the glioma microenvironment.^8^ However, the role of these Fn14-expressing cells and their relation to receptor-autonomous signaling in glioma biology remains unknown.

Here, we investigated the individual and combined contributions of tumor- vs host-derived Fn14 expression using matched Fn14-positive and Fn14-KO models. We demonstrate that Fn14 expression in both glioma cells and non-tumor cells within the TME is associated with shifts in innate and adaptive immunity. Notably, combined depletion (glioma cells and hosts) of Fn14 abrogates these effects, improving overall survival. Translational validation of these findings in GBM patient datasets revealed that Fn14-high tumors are associated with enriched signatures of immunosuppressive myeloid cells, cytokines, and checkpoint genes and exhibit significant differential responsiveness to the immune checkpoint inhibitor (ICI) pembrolizumab.

## Methods

### Cells

GL261-luciferase and CT-2A cells were provided by Dr. Michael Lim (Stanford Medicine) and Dr. Gavin Dunn (Massachusetts General Hospital, Harvard Medical School), respectively. Luciferase-expressing lines were used for all studies (see Supplementary Methods).

### CRISPR-Cas9 Gene Editing

Fn14-positive glioma cells were electroporated with Cas9 and single-guide RNAs targeting the mouse Fn14 (*TNFRSF12A*) gene. CRISPR-edited bulk populations were labeled with fluorescently tagged anti-Fn14 antibody. Fn14-negative cells were sorted using flow cytometry to generate single-cell clones. Fn14 KO was validated by Sanger sequencing, Western blotting and flow cytometry. At least *n* = 15 clones were screened for each cell line and 2 single cell clones with >95% Indel score (ICE score) and predicted protein KO score from each cell line were selected for downstream assays. Details are given in Supplementary Methods.

### Western Blot Analysis

Protein extraction and immunoblotting were performed as described previously (see Supplementary Methods for details and Supplementary Table S1 for complete list of antibodies).^8^ Densitometry was performed using ImageJ.^34^ Protein expression values were first normalized to GAPDH levels and presented as fold change relative to Fn14-positive cells/Fn14-wildtype mice samples.

### Human GBM Samples

Deidentified human GBM tissues (formalin-fixed paraffin-embedded) were obtained from the UMSOM Department of Pathology with prior approval from the UMSOM Institutional Research Board. Tissues were processed by the University of Maryland (UMB) Pathobiology Biorepository Shared Services (PBSS).^15^

### Animals

C57BL/6 (Fn14-wildtype; (Fn14-WT)) mice were purchased from Taconic Biosciences. C57BL/6 Fn14-KO mice were obtained from Dr. Linda Burkly (Biogen Inc.).^35^^,^^36^ All studies included age-matched (6-8 weeks old) mice. Male and female mice were used as available. All animal procedures were approved by the UMB Institutional Animal Care and Use Committee (IACUC) and the Office of Animal Welfare Assurance.

### Hematological Profiling of Fn14-WT and Fn14-KO Mice

Fn14-KO mice and their WT littermate controls (*n* = 5 each) were anesthetized with 4% chloral hydrate (400 mg/kg intraperitoneally), and blood was obtained via transcardial puncture for cell count with differential analysis. All procedures were conducted with the approval of the IACUC, Emory University, Atlanta, GA.

### Intracranial Implantation of Glioma Cells

Intracranial injection of glioma cells was performed as described in Supplementary Methods. Mice were routinely monitored for signs of pain and neurological distress. Body weights were recorded 3 times a week. Animals were euthanized upon more than 20% loss from initial body weight or met other pre-determined alternative endpoint criteria.

### Bioluminescence Imaging

Intracranial tumor growth was monitored via bioluminescence imaging (BLI) using the Xenogen IVIS imaging system (see Supplementary Methods). Tumor burden was estimated by calculating the total photon flux (photons/sec) within identical regions of interest drawn around the brain using Living Image software (PerkinElmer).

### Histology

Animals were euthanized, perfused with ice-cold phosphate buffered saline (PBS), followed by 4% paraformaldehyde at the appropriate endpoint. Tissues were rapidly extracted and processed for histology as described previously.^8^ Hematoxylin and Eosin (H&E) staining was performed by NDB Bio Laboratories or us using the Hematoxylin and Eosin stain kit (Vector Laboratories, H-3502). Slides were scanned using Aperio Scanscope at 20× magnification. Details are given in Supplementary Methods.

### Immunohistochemistry

Immunohistochemistry (IHC) for F4/80 was performed by NDB Bio Laboratories or in-house. CD3 staining was performed by the UMB PBSS core (see Supplementary Methods). Slides were scanned using Aperio Scanscope at 20X magnification. Positive staining for F4/80 was quantified using the positive pixel count macro from Aperio Imagescope software. Positive staining for CD3 was evaluated by a board-certified neuropathologist in a blinded fashion.

### Immunofluorescence

Brain tumor tissues were processed and co-labeled with anti-Fn14 and anti-Iba1 antibodies as described previously (see Supplementary Methods).^8^ Images were acquired on the Nikon W-1 spinning disk confocal microscope (Nikon).

### Flow Cytometry

Tumor-bearing mice were euthanized and perfused with ice-cold PBS. Brains were rapidly extracted and processed for flow cytometry (see Supplementary Methods for details and Supplementary Table S1 for complete list of antibodies).

### Bioinformatics

Human GBM single-cell RNA-sequencing (scRNA-seq) data (GSE84465)^37^ were downloaded from http://gbmseq.org and analyzed to identify Fn14-expressing cell clusters. GBM (IDH-WT) samples from syn52256654^38^ dataset were classified into Fn14-high and Fn14-low groups, and Gene Set Enrichment Analysis (GSEA), single-sample GSEA (ssGSEA), CIBERSORTx and correlation analysis were performed. GBM (IDH-WT) patients in the adjuvant only pembrolizumab-treated group from GSE121810^39^^,^^40^ dataset were classified into Fn14-high and Fn14-low groups and survival analysis was performed. No new datasets were generated in this study. Details are given in Supplementary Methods.

### Statistics

Data are presented as the mean ± SEM unless specified otherwise. Kaplan-Meier survival curves were compared using Log-rank test, with median survival times assessed using Mann-Whitney *U* test. Unpaired Student’s *t*-test or Wilcoxon rank-sum test was used to compare data from 2 groups unless specified otherwise. Mixed-effect model was used to analyze paired data. One-way ANOVA or Kruskal-Wallis test with appropriate post hoc test was used for multiple comparisons. *P* < .05 was considered statistically significant unless indicated otherwise. Statistical analyses were performed, and graphs were created using GraphPad Prism 10.0 or R. See respective figure legends and Supplementary Methods for additional details.

## Results and Discussion

### Both Neoplastic and Non-Neoplastic Cell Types Express Fn14 in Human GBM and Mouse Models of High-Grade Glioma

**Figure 1.**
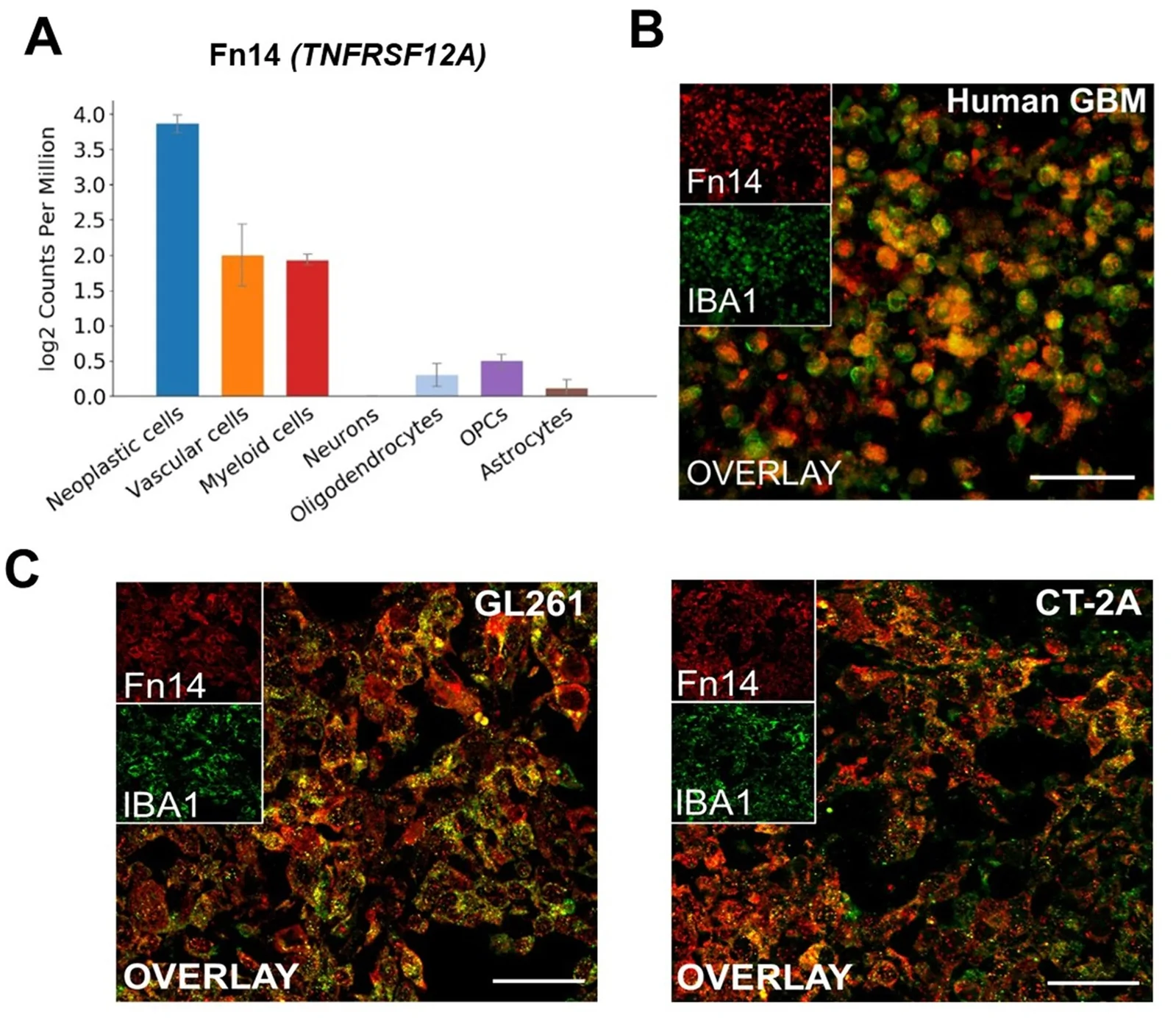
Transcriptomic analysis and immunofluorescence labeling identifies Fn14-expressing neoplastic and non-neoplastic cell types in human GBM tumors and mouse models of high-grade glioma. (A) Cell type-specific Fn14 (*TNFRSF12A*) gene expression levels analyzed by single-cell RNA-sequencing. Data sourced from publicly available single-cell RNA-seq dataset GSE84465. (B-C) Immunofluorescence co-labeling of Fn14 (red) and Iba1 (green, tumor-associated microglia/macrophages) in (B) human GBM tumor sample and (C) GL261 and CT-2A mouse glioma samples shows significant overlap (yellow). Scale bars: B = 100 µm, C = 50 μm.

We have previously identified TAMMs as one of the Fn14-expressing non-neoplastic cell types present in the glioma TME via transcriptomic analyses of a publicly available human GBM dataset.^8^ Here, we analyzed Fn14 (*TNFRSF12A*) gene expression by querying scRNA-seq data from the GSE84465 human GBM dataset.^37^ Cell type-specific gene expression analysis revealed 3 major Fn14-positive populations in the GBM TME: (i) neoplastic cells, (ii) vascular cells, and (iii) myeloid cells, with neoplastic cells having the highest levels of Fn14 gene expression (Figure 1A).

**Figure 2.**
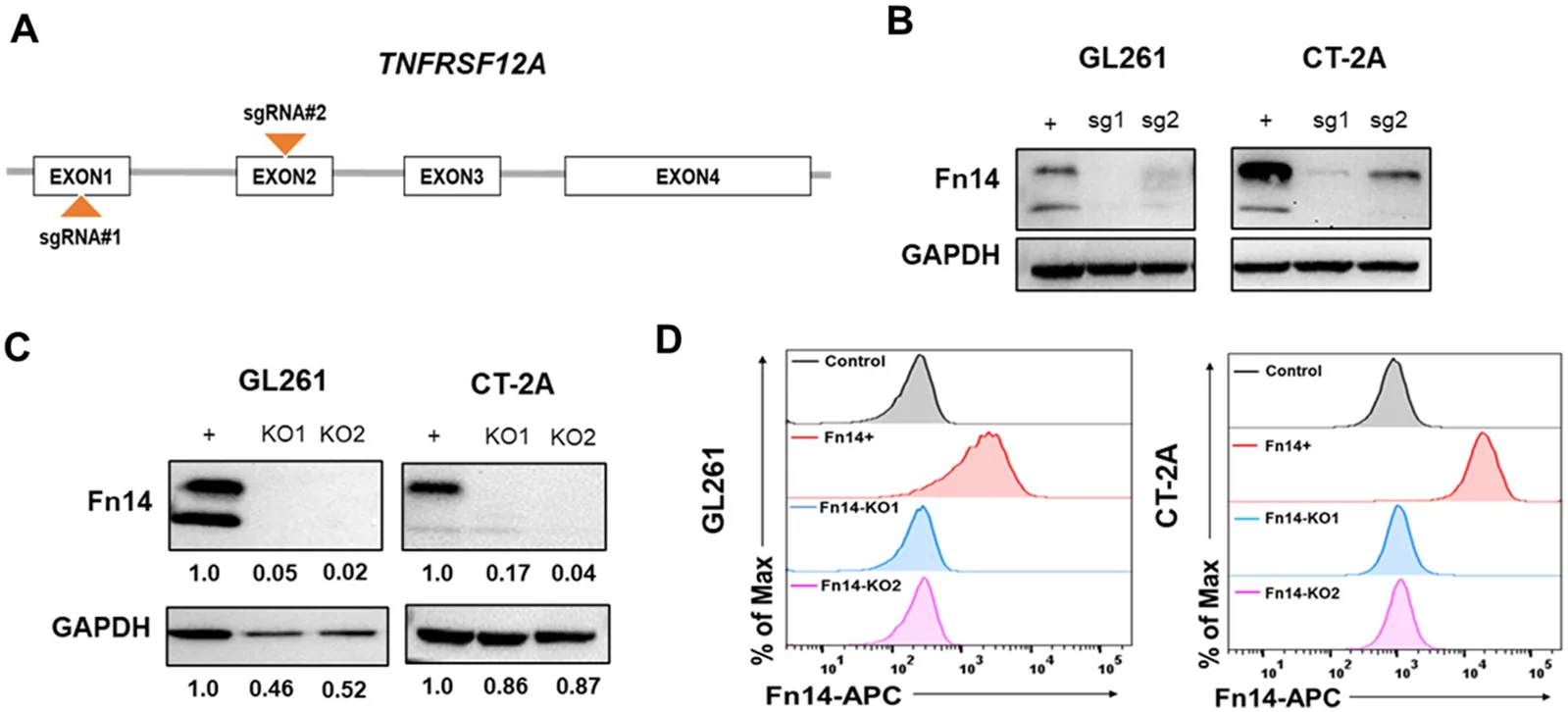
Confirmation of Fn14 knock-out in CRISPR-Cas9 edited mouse glioma cells. (A) Schematic illustrating the regions targeted by single guide RNA#1 (sgRNA#1) and single guide RNA#2 (sgRNA#2) on the *TNFRSF12A* gene. (B) Western blot analysis of Fn14 protein levels in CRISPR-Cas9-edited glioma cells following electroporation with sgRNA-Cas9 complex. (C) Western blot analysis of Fn14 protein levels in sgRNA#1-edited glioma cells following clonal selection. (D) Flow cytometric analysis of surface Fn14 expression in glioma cells after clonal selection and expansion. Isotype control is shown in the topmost row, followed by Fn14-positive cells, Fn14-KO1 cells and Fn14-KO2 cells in sequential rows.

To confirm Fn14 protein expression in myeloid cells, we performed immunofluorescence co-labeling on human GBM tissues for Fn14 and Iba1, a marker for identifying TAMMs. We observed strong co-localization of the Fn14 and Iba1 signals (Figure 1B). Next, we assessed if this observation extended to 2 well-established syngeneic mouse models of high-grade glioma (GL261 and CT-2A).^41^^,^^42^ Using the same co-labeling strategy, we analyzed brain tumor tissues from C57BL/6 mice implanted with these cell lines. Both these lines exhibit high Fn14 levels (see Figure 2B-D). Consistent with the human GBM samples, we found robust co-localization of Fn14 and Iba1 in the mouse glioma tissues (Figure 1C). Together, these results indicate that there are Fn14-expressing TAMMs in the glioma microenvironment.

Multiple high-dimensional analyses of human GBM tumors and preclinical glioma models highlight the pro- or anti-tumor roles of various stromal cells (eg, immune cells, fibroblasts, neurons, vascular cells, astrocytes, etc.) in GBM.^29^^,^^31^^,^^32^^,^^43^ Dissecting these tumor-stromal interactions is critical to identifying new therapeutic targets. Expanding our current understanding of Fn14 expression in GBM,^8^^,^^31^^,^^32^ here we confirmed vascular and myeloid cells as 2 Fn14-positive stromal cell types in the TME using scRNA-seq data from a human GBM dataset.^37^ Furthermore, we experimentally validated Fn14 expression in TAMMs, the most abundant immune cells in the GBM TME and key drivers of immunosuppression in gliomas.^44^^,^^45^

### Fn14 Loss in Both Tumor and Host Cells Extends Survival in GL261 but Not CT-2A Glioma-Harboring Mice

GL261 and CT-2A glioma models both replicate certain genetic and molecular features of human GBM, yet they differ markedly in their immune microenvironments, which is known to significantly influence experimental outcomes.^41^^,^^42^^,^^46^^,^^47^ GL261 gliomas are considered immunologically active or “hot” due to a high tumor mutational burden (TMB), functional antigen-presenting cells and a less exhausted T-cell compartment, allowing for more favorable responses to various ICIs.^42^^,^^46–51^ In contrast, CT-2A tumors are considered immunologically inert or “cold” due to a low TMB, impaired antigen presentation, and higher regulatory (Tregs) and terminally exhausted CD4+ and CD8+ T-cells.^42^^,^^46–49^^,^^51–53^ Thus, CT-2A tumors more closely resemble the immunosuppressive microenvironment of human GBM tumors.

To explore the impact of tumor- vs host-derived Fn14 expression on tumor growth and animal survival, we first generated Fn14-KO glioma cells using CRISPR-Cas9 gene editing. Fn14-positive GL261 and CT-2A glioma cells were electroporated with sgRNAs targeting exon 1 (sgRNA#1) or exon 2 (sgRNA#2) of the mouse *TNFRSF12A* gene (Figure 2A). Electroporated cells were expanded, followed by Sanger sequencing and immunoblotting to confirm KO efficiency. sgRNA#1-edited bulk populations from both lines had greater KO efficiency (Figure 2B) and were used to generate single-cell clones for further experiments. Two single-cell clones with >95% indel score and protein KO score from each cell line, as determined by Inference of CRISPR Edits (ICE) analysis of Sanger sequences were selected for downstream assays. Fn14 protein knockdown in selected single cell clones was confirmed by western blotting (Figure 2C) and flow cytometry (Figure 2D).

**Figure 3.**
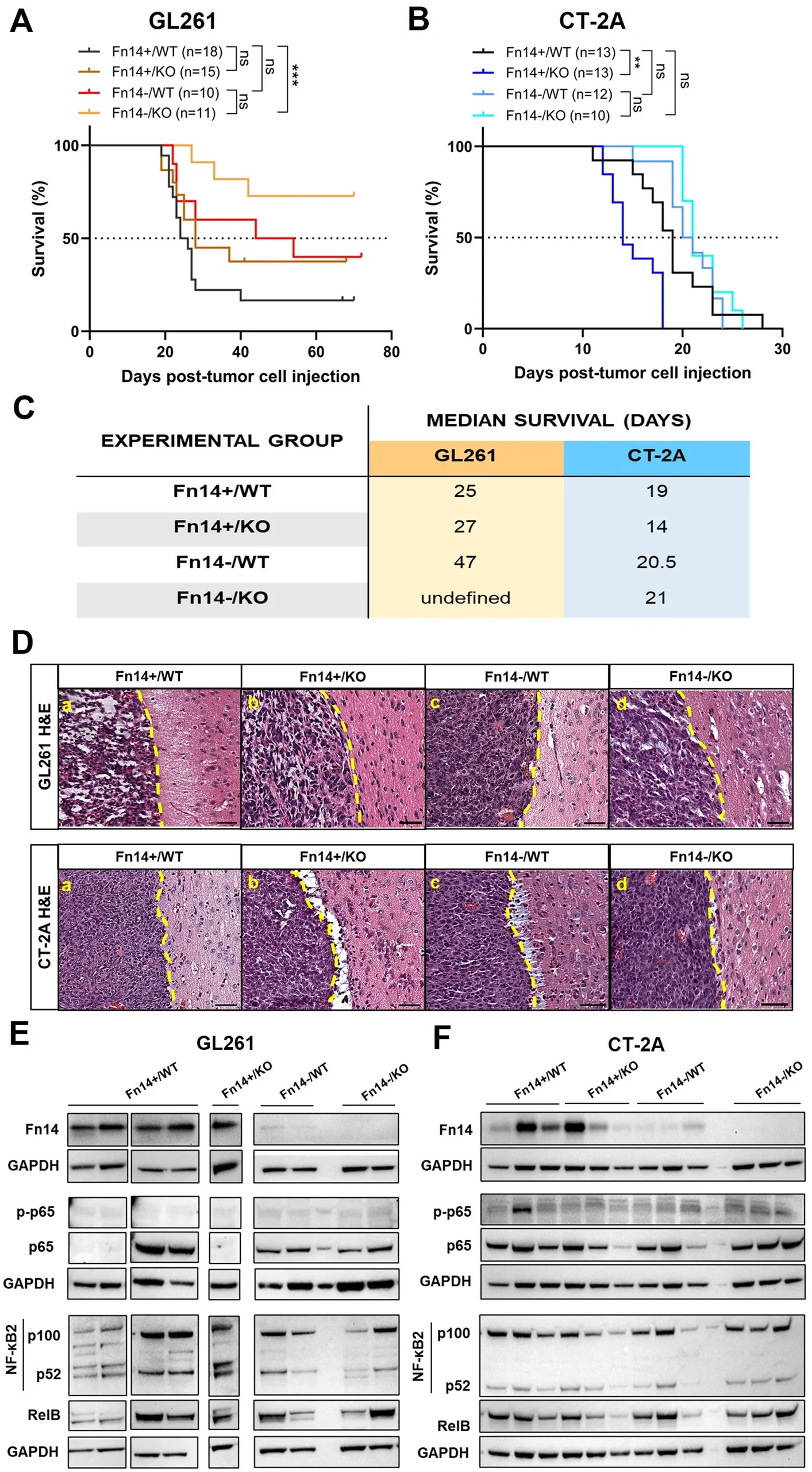
Fn14 knock-out in tumor cells and host cells has a differential impact on animal survival in GL261 and CT-2A models. (A) Kaplan-Meier survival curves for Fn14-wildtype (WT) and Fn14-knockout (KO) mice orthotopically injected with GL261 Fn14-positive or Fn14-KO glioma cells. Survival curves represent data combined from multiple cohorts. Log-rank Mantel-Cox test was used to determine statistical significance at *P* < .05 (***P < .001). (B) Kaplan-Meier survival curves for Fn14-WT and Fn14-KO mice orthotopically injected with CT-2A Fn14-positive or Fn14-KO glioma cells. Survival curves represent data combined from multiple cohorts. Log-rank Mantel-Cox test was used to determine statistical significance at *P *< .05 (**P < .01). (C) Median survival times for Fn14-WT and Fn14-KO mice with Fn14-positive and Fn14-KO GL261 and CT-2A tumors. (D) Histological evaluation of GL261 and CT-2A tumors from all 4 Fn14+/- tumor-host pairings collected at survival endpoint indicates no difference in brain invasion. Yellow stippled line represents tumor margins. Scale bar = 50μm. (E-F) Western blot analysis of Fn14 and NF-κB pathway protein expression levels in brain tumor samples collected at survival endpoint from all 4 Fn14+/- tumor-host pairings. Lanes represent samples collected from independent animals. Results from (E) GL261 and (F) CT-2A tumors.

We then established intracranial tumors using GL261 and CT-2A Fn14-positive or Fn14-KO glioma cells in syngeneic Fn14-WT or Fn14-KO C57BL/6 mice. KO#1 clone from both cell lines was used for all in vivo experiments. Tumor-take rate and growth were monitored by BLI. Histological and hematological analysis revealed no significant differences in the overall tissue structure and blood cell composition of Fn14-WT and -KO mice (Supplementary Figure S1, Supplementary Table S2). Fn14-WT mice with Fn14-KO GL261 tumors (Supplementary Figure S2A) and Fn14-KO mice with Fn14-KO CT-2A tumors (Supplementary Figure S2B) had the lowest tumor take rates. The median survival for Fn14-WT mice with Fn14-positive (Fn14+/WT) GL261 tumors was 25 days (Figure 3A). We observed that Fn14 deletion in hosts alone (Fn14+/KO) had no significant impact on overall survival compared to Fn14+/WT controls (median survival = 27 days vs 25 days, respectively, *P* = .264) (Figure 3A). Fn14 KO in GL261 tumor cells alone (Fn14-/WT) resulted in a ∼1.9-fold improvement in survival compared to the Fn14+/WT group. Spontaneous tumor clearance was observed in 40% of animals from this cohort (median survival = 47 days vs 25 days, respectively, *P* = .178) (Figure 3A). Finally, Fn14 KO in both tumor cells and hosts (Fn14-/KO) significantly increased overall survival compared to Fn14+/WT controls (median survival = undefined vs 25 days, *P* < .001) (Figure 3A). Spontaneous tumor clearance was observed in more than half (∼72%) of the animals in the GL261 Fn14-/KO cohort (Figure 3A). BLI data for GL261 tumor-bearing mice mirrored the survival data trends (Supplementary Figure S3). Animals from all 4 groups had a comparable tumor burden at one-week post-injection (Supplementary Figure S3). However, over time, a gradual reduction in tumor luminescence was observed for the Fn14-/KO cohort. Ultimately, 8 out of 11 mice achieved complete tumor resolution, surviving until the study endpoint (Supplementary Figure S3A and B).

The median survival for CT-2A Fn14+/WT group was 19 days (Figure 3B). Unlike GL261 gliomas, we observed that Fn14 KO in hosts alone (Fn14+/KO) significantly reduced overall survival compared to Fn14+/WT controls (median survival = 14 days vs 19 days, respectively, *P* = .0028) (Figure 3B). Tumor cell luminescence measurements for the Fn14+/KO group were consistently higher, suggesting a rapid tumor progression rate compared to other groups (Supplementary Figure S4A and B). Comparison of tumor cell luminescence on day 15 post-injection revealed that Fn14+/KO group had a significantly higher tumor burden compared to other groups (Supplementary Figure S4C). Fn14-/WT and Fn14+/WT groups had comparable survival rates (median survival = 20.5 days vs 19 days, respectively, *P* = 0.333), indicating that Fn14 KO in tumor cells alone has no significant impact on survival (Figure 3B). Finally, we observed that Fn14 KO in both tumor cells and hosts (Fn14-/KO) substantially reduced mortality risk at early time points for CT-2A glioma-bearing mice. Still, the overall survival of the Fn14-/KO group was minimally altered compared to the Fn14+/WT controls (median survival = 21 days vs 19 days, *P* = .170) (Figure 3B). Median survival times for all cohorts are summarized in Figure 3C. Together, these results offer compelling evidence that Fn14 expression in both tumor and host cells contributes to glioma progression.

### Fn14 KO in Tumor Cells, Host Cells or Both Has Minimal Impact on Tumor Histology, Immune Cell Infiltration, and NF-κB Signaling

We previously demonstrated that Fn14 overexpression in rat gliomas enhances tumor invasiveness, pseudopalisading necroses, TAMM infiltration and non-canonical NF-κB signaling.^8^ To evaluate how Fn14 depletion affects glioma histopathology, we performed H&E staining on brain tumor tissues collected at the survival endpoint from all 4 Fn14 tumor-host pairings for both glioma models. Samples were blinded and evaluated for cellular atypia, invasive margins, necrosis, and microvascular proliferation by a board-certified neuropathologist. No striking differences were observed in the histological features between Fn14-positive or Fn14-KO tumors across all host backgrounds (Supplementary Figure S5). GL261 tumors showed frequent microcystic changes and low lymphocyte levels (Supplementary Figure S5A) while CT-2A tumors were hypercellular with spindle-like mesenchymal appearance, lacking microcystic changes but still exhibiting low lymphocyte levels (Supplementary Figure S5B). All tumors from both models exhibited well-circumscribed margins with minimal invasion into adjacent normal brain parenchyma (Figure 3D). Thus, unlike our previous findings where we overexpressed Fn14,^8^ endogenous Fn14 levels had no significant impact on tumor invasiveness.

Given previous reports that TWEAK/Fn14 signaling promotes TAMM infiltration in cholangiocarcinoma^20^ and rat gliomas,^8^ we performed IHC for the macrophage marker F4/80. We found no significant differences in the density of F4/80+ cells in the GL261 (Supplementary Figure S6A and C) or CT-2A (Supplementary Figure S6B and D) models. However, Fn14-/KO tumors from both models had overall decreased F4/80+ macrophages, suggesting a modest contribution of Fn14 to TAMM recruitment (Supplementary Figure S6C and D). We also stained for CD3 to assess total T-cell infiltration. Overall, Fn14-/KO tumors from both models had the lowest central and peripheral CD3+ T-cell frequencies (Supplementary Figure S7). In GL261 tumors, even Fn14+/KO tumors exhibited a significant reduction in CD3+ T-cells compared to Fn14+/WT tumors (Supplementary Figure S7A and C). No such differences in CD3+ T-cell density occurred in CT-2A tumors across all genotypes (Supplementary Figure S7B and D).

Finally, we performed Western blot analysis to assess the status of the canonical and non-canonical NF-κB, MAPK, Akt, and JAK/STAT signaling pathways, as well as Fn14 expression levels, in all endpoint brain tumor tissues. Minimal to no Fn14 expression was detected in Fn14-/WT and Fn14-/KO tissues for both models, thus confirming that the Fn14-KO mice do not express Fn14 and that Fn14 KO in tumor cells is maintained in vivo (Figure 3E and F). Unlike our prior observations from Fn14-high vs Fn14-low rat gliomas,^8^ we found no significant differences in either canonical or non-canonical NF-κB signaling across any group in either glioma model (Figure 3E and F, Supplementary Figure S8). Similarly, we did not detect any changes in the activity of other signaling pathways in any of the 4 tumor types from both models (Supplementary Figure S9). These findings suggest that the impact of Fn14 expression on survival appears to be independent of its effects on glioma cell invasion and the activity of various signaling cascades.

### Fn14 Depletion in Tumor and Host Cells Reduces Tumor-Supporting TAMM Infiltration

**Figure 4.**
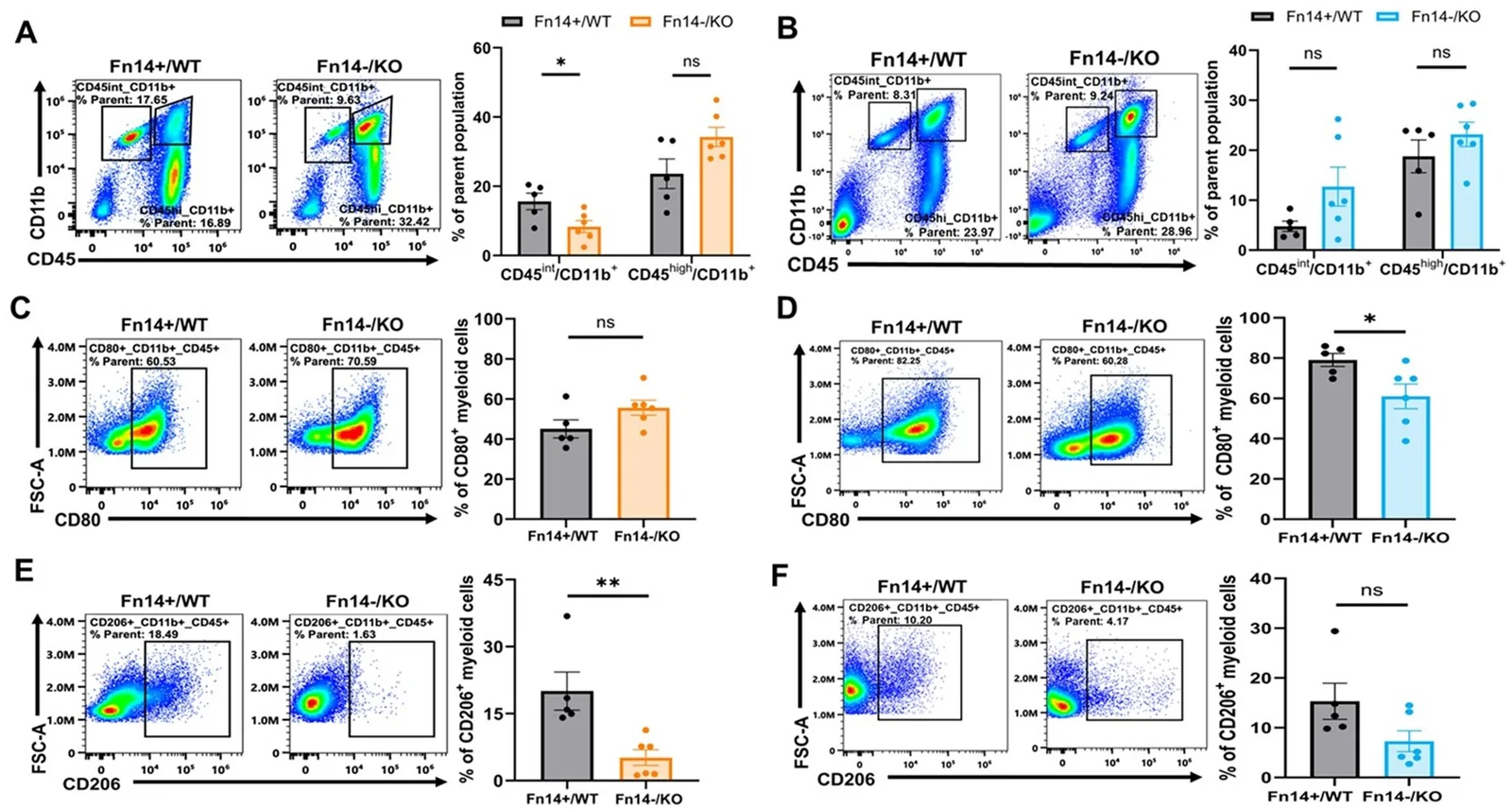
Fn14 depletion from tumor cells and host cells reduces infiltration of tumor-supporting CD206+ macrophages in GL261 and CT-2A gliomas. Fn14-WT and Fn14-KO mice were orthotopically injected with Fn14-positive and Fn14-KO glioma cells, respectively. Two weeks post-injection, mice were euthanized, brain tumor tissue was collected and processed for flow cytometric analysis of tumor infiltrating immune cells. (A-B) Percentage of microglia (CD45int CD11b+) and bone marrow-derived macrophages (BMDMs) (CD45high CD11b+) in (A) GL261 and (B) CT-2A Fn14+/WT and Fn14-/KO tumors. (C-D) Percentage of M1-like macrophages (CD45high CD11b+ CD80+) for (C) GL261 and (D) CT-2A Fn14+/WT and Fn14-/KO tumors. (E-F) Percentage of M2-like macrophages (CD45high CD11b+ CD206+) in (E) GL261 and (F) CT-2A Fn14+/WT and Fn14-/KO tumors. Sample sizes: Fn14+/WT, *n* = 5 and Fn14-/KO, *n* = 6 for both GL261 and CT-2A models. Unpaired Student’s *t*-test or Mann-Whitney U test was used to determine statistical significance at *P* < .05 (*P < .05, **P < .01). Data are presented as mean ± SEM.

Although both models showed similar trends in TAMM and total T-cell infiltration following Fn14 depletion, only GL261 tumors exhibited a statistically significant improvement in survival. Considering these results and the known differences in the immune TME of GL261 and CT-2A gliomas,^41^^,^^42^^,^^46–53^ we postulated that in the more immunologically active GL261 tumors, Fn14 KO enhances anti-tumor immunity and tumor clearance, while in the more immunosuppressed CT-2A model, Fn14 loss alone may be insufficient to overcome the intrinsic immune resistance. To test this hypothesis, we performed immunophenotyping of GL261 and CT-2A Fn14+/WT and Fn14-/KO tumors. Two weeks post-tumor cell injection, tumor-bearing animals from both groups were euthanized, and brains were harvested for flow cytometric analysis to characterize tumor-infiltrating immune cells. We focused on 3 innate immune populations: dendritic cells (DCs) (CD45+ CD11c+), microglia (CD45-intermediate, int, CD11b+), and bone marrow-derived macrophages (BMDMs) (CD45-high CD11b+) (gating strategy in Supplementary Figure S10A). Fn14 KO had no effect on DC distribution in either GL261 (Supplementary Figure S11A) or CT-2A (Supplementary Figure S11B). GL261 Fn14-/KO tumors had significantly fewer microglial cells and a modest increase in BMDM infiltration compared to Fn14+/WT controls (Figure 4A). No such differences were seen in CT-2A tumors (Figure 4B).

TAMMs dominate the glioma immune landscape.^43–45^ While the M1/M2 classification oversimplifies their heterogeneity, glioma TAMMs typically adopt an M2-like, tumor-supportive phenotype that promotes immunosuppression and tumor progression.^44^^,^^45^^,^^54^ To evaluate the impact of Fn14 on TAMM polarization, we assessed M1-like (CD80) and M2-like (CD206) CD45+ CD11b+ myeloid populations. Fn14-KO had no significant effect on CD80+ TAMMs in GL261 tumors (Figure 4C). However, there was a significant decrease in CD80+ TAMM infiltration in CT-2A Fn14-/KO tumors compared to Fn14+/WT controls (Figure 4D). M2-like CD206+ TAMMs were significantly reduced in GL261 Fn14-/KO tumors compared to Fn14+/WT controls (Figure 4E). Although not significant, a marked reduction in CD206+ TAMMs was also noted in CT-2A Fn14-/KO tumors compared to Fn14+/WT controls (Figure 4F). Thus, Fn14 KO in both tumor and host cells reduces the recruitment of tumor-supporting M2-like TAMMs in both GL261 and CT-2A gliomas.

### Fn14 Depletion in Both Tumor and Host Cells Impacts Exhausted T-Cell Populations in GL261 but Not CT-2A Gliomas

**Figure 5.**
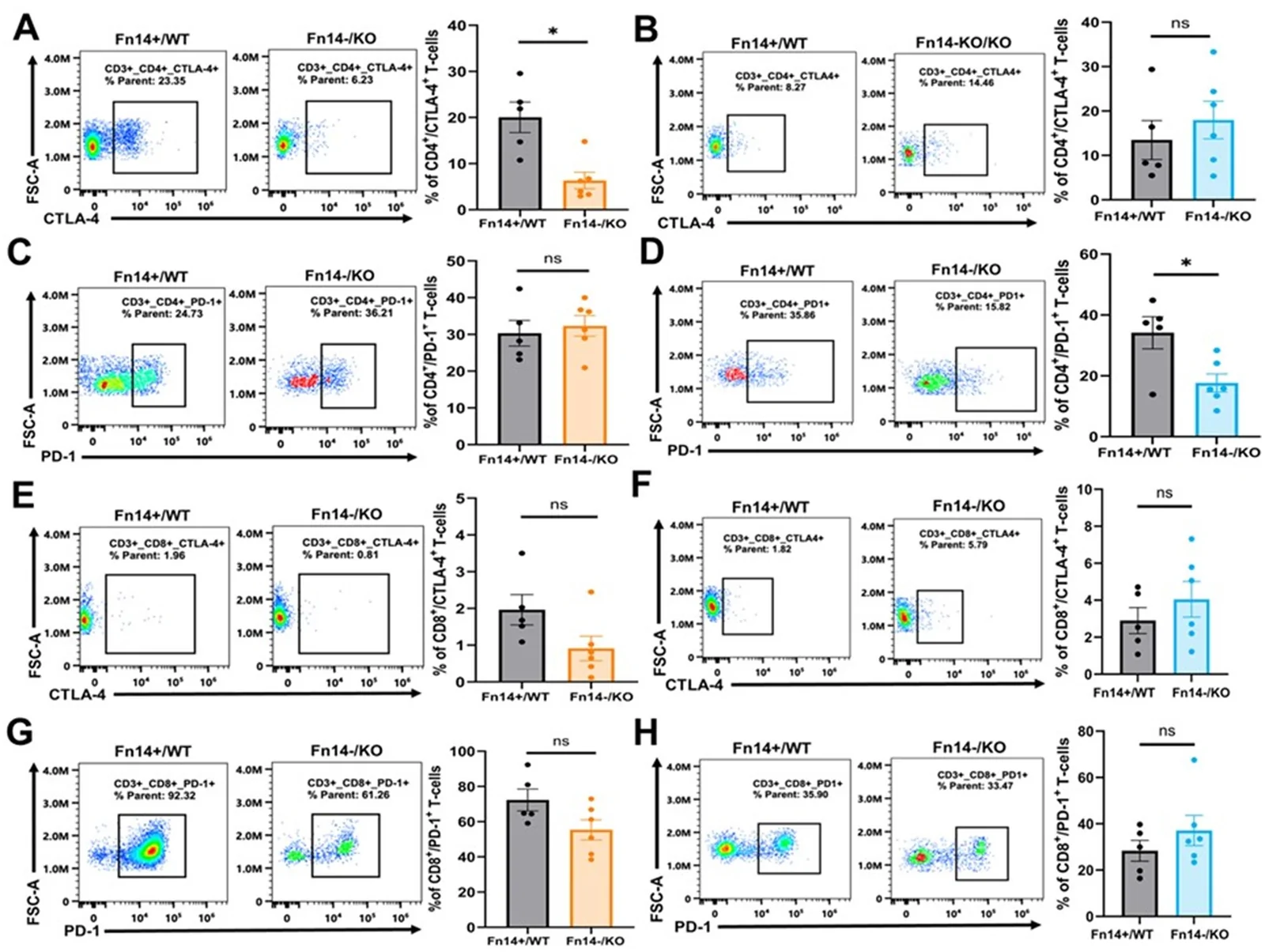
Eliminating Fn14 from tumor cells and host cells reduces the relative frequency of exhausted T-cells in GL261 but not CT-2A gliomas. Fn14-WT and Fn14-KO mice were orthotopically injected with Fn14-positive and Fn14-KO glioma cells, respectively. Two weeks post-injection, mice were euthanized, brain tumor tissue was collected and processed for flow cytometry analysis of tumor infiltrating immune cells. (A-B) Percentage of CTLA4+ CD4+ T-cells in (A) GL261 and (B) CT-2A Fn14+/WT and Fn14-/KO tumors. (C-D) Percentage of PD-1+ CD4+ T-cells in (C) GL261 and (D) CT-2A Fn14+/WT and Fn14-/KO tumors. (E-F) Percentage of CTLA4+ CD8+ T-cells in (E) GL261 and (F) CT-2A Fn14+/WT and Fn14-/KO tumors. (G-H) Percentage of PD-1+ CD8+ T-cells in (G) GL261 and (H) CT-2A Fn14+/WT and Fn14-/KO tumors. Sample sizes: Fn14+/WT mice, *n* = 5 and Fn14-/KO mice, *n* = 6 for both GL261 and CT-2A models. Unpaired Student’s *t*-test or Mann-Whitney *U* test was used to determine statistical significance at *P* < .05 (*P < .05). Data are presented as mean ± SEM.

To determine whether Fn14 expression influences the adaptive immune compartment in gliomas, we assessed the relative abundance of CD4+ helper T-cells and CD8+ cytotoxic T-cells expressing the exhaustion markers CTLA-4 and PD-1 (gating strategy in Supplementary Figure S10B). Consistent with our CD3 IHC data, we observed that overall Fn14-/KO tumors had reduced CD3+ T-cell infiltration compared to Fn14+/WT controls, which was statistically significant in GL261 (Supplementary Figure S11C) but not CT-2A (Supplementary Figure S11D) tumors. Fn14 KO affected T-cell exhaustion markers differently across the 2 models. In the GL261 model, Fn14-/KO tumors had a significant reduction in CTLA-4+ CD4+ T-cells compared to Fn14+/WT controls (Figure 5A). No significant differences were observed in CT-2A gliomas (Figure 5B). PD-1+ CD4+ T-cells were unchanged in GL261 tumors (Figure 5C) but significantly decreased in Fn14-/KO CT-2A tumors (Figure 5D). Fn14 KO had a more pronounced impact on exhausted CD8+ T-cells in GL261 gliomas. Although not significant, Fn14-/KO GL261 tumors showed a consistent downward trend in both CTLA-4+ CD8+ (Figure 5E) and PD-1+ CD8+ T-cells (Figure 5G) compared to Fn14+/WT controls. In contrast, an increasing trend in the relative frequency of both CTLA-4+ CD8+ T-cells (Figure 5F) and PD-1+ CD8+ T-cells (Figure 5H) was observed in Fn14-/KO CT-2A tumors compared to Fn14+/WT controls. Taken together, our results suggest that Fn14 co-deletion in tumor cells and hosts is more effective at reducing the overall abundance of exhausted T-cells in the immunologically active GL261 gliomas compared to the immunologically inert CT-2A gliomas.

### Elevated Fn14 Expression in Human GBM Tumors Correlates with Immunological Shifts, Increased M2-like Macrophage Abundance and Poor Clinical Response to Anti-PD-1 Therapy

**Figure 6.**
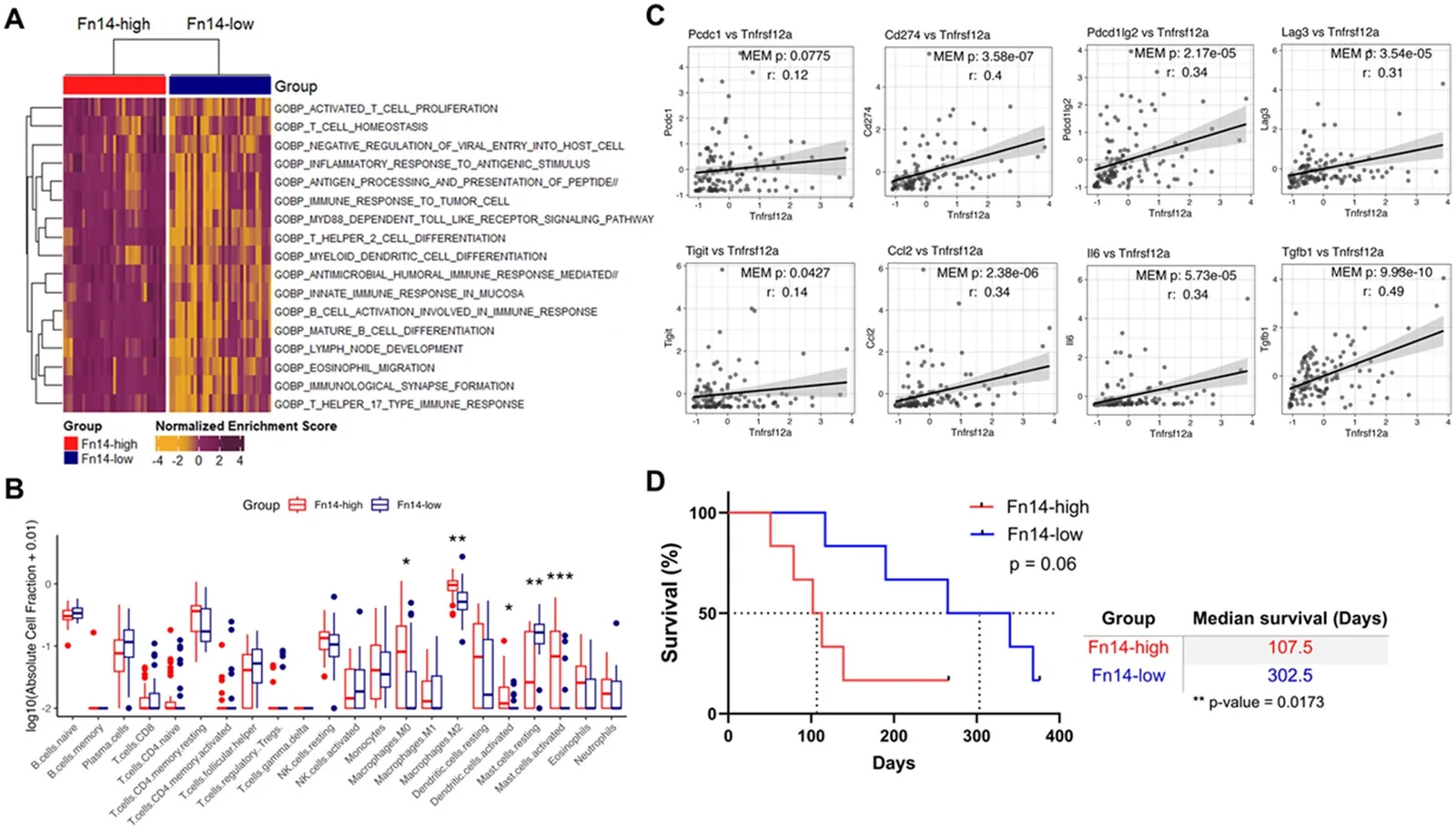
Elevated Fn14 expression in human GBM tumors is associated with immunosuppressive features and poor patient response to immune checkpoint inhibitors. (A) Heat map represents the immune response-associated biological processes upregulated in Fn14-high samples following single sample Gene Set Enrichment Analysis (ssGSEA) of IDH-WT human GBM tumors from syn52256654 dataset. All biological processes have an adjusted *P*-value < .05 with Mann-Whitney *U* test of enrichment scores between Fn14-high and Fn14-low samples. No biological processes were significantly enriched in Fn14-low samples after redundancy filtering using REVIGO. (B) Immune cell abundance in Fn14-high (red, top quartile, *n* = 31) vs Fn14-low (blue, bottom quartile, *n* = 31) IDH-WT GBM tumors from syn52256654 dataset assessed using CIBERSORTx algorithm. Log-transformed absolute fraction values for each cell type are graphed. A Wilcoxon rank sum test was performed for each cell type (*P < .05, **P < .01, ***P < .001). (C) Correlation plots with linear fit lines for Fn14 and select immune response-associated genes. Pearson correlations “*r*” and mixed-effect model (MEM) *P*-values are plotted for each gene pair. Shaded regions represent 95% confidence intervals. *P* < .05 was considered statistically significant. (D) Kaplan-Meier survival analysis of anti-PD-1 immunotherapy outcomes in GBM patients with Fn14-high (red) vs Fn14-low (blue) tumors. Overall survival data for IDH-WT GBM patients treated with anti-PD-1 monoclonal antibody pembrolizumab in adjuvant only paradigm from GSE12810 dataset was analyzed. Median Fn14 expression was used to stratify patients into Fn14-high and Fn14-low groups. Median survival for Fn14-high group (red, *n* = 6) and Fn14-low group (blue, *n* = 6) was 107.5 days and 302.5 days, respectively. Log-rank Mantel-Cox test was used to determine statistical significance at *P* < .05. Mann-Whitney *U* test was used for direct comparison of median survival times for Fn14-high vs Fn14-low groups.

To determine if there were any Fn14-associated changes in the human GBM immune microenvironment, we mined a human GBM tumor RNA-sequencing database.^38^ We first performed GSEA on IDH-WT Fn14-high vs Fn14-low tumors using Gene Ontology, focusing on ‘immune process’ terms. Several pathways linked with both innate and adaptive immunity like leukocyte migration, antigen processing, and T-cell/macrophage activation, were significantly enriched in the Fn14-high tumors. Pathways related to erythrocyte, natural killer cell, and thymus development were enriched in the Fn14-low tumors, but these changes were not statistically significant (Supplementary Figure S12A and B). ssGSEA analysis revealed similar trends, with Fn14-high tumors showing significant enrichment for pathways related to T-cell and B-cell activation, antigen processing, inflammatory responses and myeloid cell functions (Figure 6A). To assess if Fn14 expression in human GBM tumors impacts immune cell distribution, we performed CIBERSORTx analysis. We found distinct differences, primarily in the innate immune cell compartment, between Fn14-high vs Fn14-low groups (Figure 6B). M2-like macrophages were the most abundant immune cell type and were significantly enriched in the Fn14-high tumors (Figure 6B), consistent with our immunophenotyping data (Figure 4E and Figure 4F). Fn14-high tumors also exhibited significantly higher numbers of M0 macrophages, activated DCs and activated mast cells compared to Fn14-low tumors (Figure 6B). A significant positive correlation was noted between Fn14 gene expression and several immune checkpoint genes (*CD274, PDCD1LG2, LAG3, TIGIT*) and immunosuppressive cytokine genes (*CCL2, IL-6, TGF-β*) (Figure 6C). To explore the possible clinical relevance of these findings, we examined survival outcomes for GBM patients with either Fn14-high or Fn14-low tumors treated with the anti-PD-1 antibody pembrolizumab.^39^^,^^40^ Only IDH-WT GBM patients from the adjuvant group who received the anti-PD-1 treatment post-surgery were included in the analysis to account for the immunological changes and survival differences induced by neoadjuvant anti-PD-1 administration.^39^^,^^40^ We found that the Fn14-high group had a remarkably shorter overall survival compared to the Fn14-low group (median survival of 107.5 vs 302.5 days, respectively) (Figure 6D). This finding is consistent with computational analyses of patient datasets implicating the TWEAK/Fn14 signaling pathway as an important driver of immunosuppression and immune checkpoint therapy resistance in various solid tumors, including head and neck cancer,^27^^,^^28^ cervical cancer,^27^ gastric cancer,^23^^,^^55^ lung cancer,^24^ and osteosarcoma.^56^ In similar glioma-focused bioinformatics studies, Fn14 gene expression has been associated with increased immunosuppression,^31–33^ tumor-supporting M2-like macrophage recruitment,^31–33^ and regulatory T-cells,^31^^,^^33^ as well as poor response to immune checkpoint inhibitors.^31^ Collectively, our findings not only reinforce these prior computational predictions but further strengthen the idea that high Fn14 expression may promote immunosuppression and influence immunotherapy outcomes in GBM patients. Specifically, inhibiting Fn14 expression or its activity may be a potential therapeutic strategy to prime immunologically cold tumors like GBMs and sensitize them to ICIs.

In summary, we validated the presence of Fn14 expression in tumor cells and TAMMs in the GBM TME. We discovered that eliminating Fn14 from both glioma and host cells had a significant and differential impact on host survival. Interestingly, the greatest impact was observed in the more “immunologically active” glioma model (GL261) compared to the more ‘immunosuppressed’ model (CT-2A), suggesting that Fn14 is one part of a complex immunosuppressive landscape in gliomas. Consistent with the survival data, Fn14 depletion resulted in significantly less tumor-supporting TAMMs and an overall downward shift in exhausted T-cells in the immunologically active GL261 model compared to the immunologically inert CT-2A model. To the best of our knowledge, this study is the first to provide experimental evidence of Fn14-driven immunosuppression in experimental gliomas. Analysis of human GBM datasets revealed that high Fn14 expression correlated with elevated immunosuppressive cell phenotypes, expression of immune checkpoint markers and immunosuppressive cytokines. Furthermore, GBM patients with Fn14-high tumors showed less responsiveness to the immune checkpoint inhibitor pembrolizumab in a post hoc analysis. Together, these findings reveal a new, previously unappreciated role for Fn14 in modulating the innate and adaptive glioma immune microenvironment and support further studies testing anti-Fn14 and immunotherapy such as ICI combinations for GBM.

Several anti-TWEAK or anti-Fn14 antibodies have been tested in preclinical and clinical settings for solid tumors with variable success.^57–60^ Most of the anti-Fn14 antibodies exhibit agonistic activity. Thus, these antibodies, as well as anti-TWEAK antibodies, will not inhibit receptor-autonomous signaling, which is the most likely mechanism of Fn14-mediated effects in Fn14-high tumors.^9^^,^^10^^,^^13^ Indeed, TWEAK-independent Fn14 signaling was first described in Brown et al, where they showed that HEK293 cells overexpressing mutant Fn14 receptors unable to bind TWEAK can still activate NF-κB signaling.^13^ Additional studies in glioma,^4^^,^^12^ breast,^61^ lung,^62^ and gastric cancer^26^ cells have further shown that forced Fn14 expression in Fn14-low cells can initiate similar cellular responses to those observed post-TWEAK stimulation. Importantly, Johnston et al found that tumor cell-derived Fn14, but not TWEAK, exacerbated cancer-induced cachexia^21^ and blocking Fn14-driven signaling using anti-Fn14 antagonistic antibodies significantly reduced these effects.^21^

We have previously demonstrated that forced overexpression of Fn14 alone in neural precursor cells is sufficient to convert low-grade rat gliomas into highly invasive, TAMM-rich tumors.^8^ Minimal TWEAK expression was detected in these Fn14-high tumors, similar to what is observed in human Fn14-high tumors,^7^ and the levels were comparable to that in normal brain tissues.^8^ We found that Fn14+/WT GL261 tumors had lower levels of TWEAK (data not shown) and significantly worse survival outcomes compared to the Fn14-/KO tumors. Notably, Fn14 deletion in both tumor cells and hosts had no evidence of impact on downstream Fn14-regulated signaling pathways. Although the exact mechanism remains unclear, the combined molecular and survival data strongly support a role for TWEAK-independent, receptor-autonomous activity in gliomas with high Fn14 expression. Further studies are warranted to dissect the differential components of TWEAK-dependent vs TWEAK-independent Fn14 signaling in gliomas and identify the specific signal transduction pathways involved in modulating these important effects. This work will help to identify promising therapeutic opportunities to block receptor-autonomous Fn14 signaling, mitigate the pro-tumorigenic, immunosuppressive effects, and improve patient outcomes, especially in combination with checkpoint inhibitor therapies.

The study has some notable limitations. First, we utilized a global Fn14-KO mouse model and therefore did not evaluate tissue- or cell type-specific Fn14 effects. Second, although combined Fn14 depletion was found to improve survival, Fn14 KO in hosts alone also decreased survival in the CT-2A model, reinforcing prior results when we overexpressed Fn14 in early gliomagenesis using the RCAS/tv-a system.^8^ These findings indicate complex underlying immunosuppressive mechanisms that merit further investigation. Finally, while our immunophenotyping focused on examining Fn14-mediated effects in major innate and adaptive immune cells, other known immunosuppressive cell types (eg, myeloid-derived suppressor cells, neutrophils, T-regs) in the glioma TME were not evaluated.^43^ Further functional studies are warranted to decipher the precise immunological mechanisms involved in Fn14-driven immunosuppression in gliomas. This work would offer new insights into therapeutic development and eventual clinical testing in GBM patients with Fn14-overexpressing tumors.

## Supplementary Material

vdag023_Supplementary_Data

## Data Availability

All data associated with this study are available within the paper and its Supplementary Information. No new transcriptomic datasets were generated as part of this study. Single-cell RNA-sequencing data for human GBM tumors from the GSE84465^37^ dataset was downloaded from http://www.gbmseq.org/. RNA-sequencing data for human GBM tumors from the syn52256654^38^ dataset was downloaded from https://www.synapse.org/Synapse:syn52256654. RNA-sequencing data for human GBM tumors from the GSE121810^39^^,^^40^ dataset was downloaded from Gene Expression Omnibus (GEO). Survival data for GBM immunotherapy outcomes were obtained from McFaline-Figueroa et al.^40^ All data including R scripts will be made available upon reasonable request. All correspondence and requests for data and materials should be addressed to the corresponding authors J.A.W. and G.F.W.
