# Correction to: The TNFR superfamily member Fn14 impacts immunity and survival in experimental gliomas and response to immune checkpoint inhibitor therapy in glioblastoma patients

**DOI:** 10.1093/noajnl/vdag222

**Published:** 2026-08-27

This is a correction to: Pranjali P Kanvinde, Adarsha P Malla, Alexandra A Seas, Emylee McFarland, Jennifer R Fang, Matthew J Flick, Nina P Connolly, Angad Beniwal, Nima Sharifai, Chixiang Chen, Manuel Yepes, Eli E Bar, Pavlos Anastasiadis, Nhan L Tran, Jeffrey A Winkles, Graeme F Woodworth, The TNFR superfamily member Fn14 impacts immunity and survival in experimental gliomas and response to immune checkpoint inhibitor therapy in glioblastoma patients, *Neuro-Oncology Advances*, Volume 8, Issue 1, January-December 2026, vdag023, https://doi.org/10.1093/noajnl/vdag023

In the originally published version of this manuscript, the ‘Supplementary data’ section contained multiple different versions of the supplementary data file.

This error has been corrected so that only the correct supplementary data file remains.

