# Supplementary material for: The TNFR superfamily member Fn14 impacts immunity and survival in experimental gliomas and response to immune checkpoint inhibitor therapy in glioblastoma patients
Source: Neurooncol Adv. 2026 Feb 12;8(1):vdag023. doi: 10.1093/noajnl/vdag023 (PMC12994697; doi:10.1093/noajnl/vdag023)
Supplement: vdag023_Supplementary_Data [file vdag023_supplementary_data.zip › 27-Feb-2026_074259_NAdv_Fn14_paper_suppl2.docx]

**The TNFR superfamily member Fn14 impacts immunity and survival in experimental gliomas and response to immune checkpoint inhibitor therapy in GBM patients**

Pranjali P. Kanvinde^1,2^, Adarsha P. Malla^1,2^, Alexandra A. Seas^1,2^, Emylee McFarland^1,2^, Jennifer R. Fang^1,2^, Matthew J. Flick^3,4,5^, Nina P. Connolly^1,2#^, Angad Beniwal^3,4^, Nima Sharifai^6^, Chixiang Chen^1,7^, Manuel Yepes^8,9,10^, Eli E. Bar^1,2^, Pavlos Anastasiadis^1,2,11,12^, Nhan L. Tran^3,13^, Jeffrey A. Winkles^1,2*^, Graeme F. Woodworth^1,2,11,12*^

**SUPPLEMENTARY INFORMATION:**

**Supplementary Methods:**

Cell culture

CT-2A cells were transduced with a lentivirus encoding firefly luciferase (GeneCopoeia Inc.) followed by selection using puromycin (1 µg/ml) to obtain stable luciferase-expressing cells. Luciferase-expressing GL261 and CT-2A lines were used for all studies. All cell lines were cultured in DMEM (Corning, 10-013-CV) supplemented with 10% fetal bovine serum (GIBCO, 16140-071) and 1% penicillin-streptomycin (1000 units/L, Invitrogen 15140122) in a humidified incubator (95% air, 5% CO_2_) maintained at 37⁰ C and routinely tested for mycoplasma.

Generation of Fn14 knockout (KO) glioma cells using CRISPR-Cas9 gene editing

Single guide RNAs (sgRNAs) targeting either exon1 (sgRNA#1: 5’-CCAAGCCGAATCCCAACACG-3’) or exon2 (sgRNA#2: 5’-GCTGCCGCTAGAGCATGGGG-3’) of the mouse Fn14 (*TNFRSF12A*) gene were designed using CRISPick tool from the Broad Institute (29,30) and synthesized by Synthego. The sgRNAs were incubated with Cas9 (TrueCut Cas9 Protein v2, Invitrogen, A36499) and glioma cells were electroporated with the sgRNA-Cas9 ribonucleoprotein complex with a single pulse of 1300 volts for 30 ms using the Neon Transfection system (Invitrogen). After electroporation, cells were expanded, subjected to PCR and Sanger sequencing using the following primers from IDT: sgRNA#1: forward primer, 5’-GAGCACAAATCGCAAATCCT-3’, reverse primer, 5’-TACTCGCAAAGCAGAGTGGCTG-3’; sgRNA#2: forward primer, 5’- TCTACAACCCTCCTCTCTGC -3’, reverse primer, 5’- TCACCTGCGAGGAATGAGTG -3’. Editing efficiency was confirmed by analysis of Sanger sequencing reads using Inference of CRISPR Edits (ICE) software (EditCo). Fn14 protein levels in the KO cells were confirmed by Western blot (see below). To obtain single-cell clones, single-cell suspensions of glioma cells were labeled with APC-conjugated anti-human/mouse CD266 (Fn14, TWEAK-R) antibody (Biolegend, 314107). Fn14-negative cells were sorted into 96 well plates using the BD FACS Aria sorter at 1 cell/well. Wells with single-cell clones were selected for further expansion. Editing efficiency in the clones was verified by Sanger sequencing (Genewiz) and ICE analysis (EditCo) as described above. At least n=15 clones were screened from each cell line and two single-cell clones with >95% Indel Score (ICE score) and predicted protein KO score from each cell line were selected for further downstream assays. Fn14 protein levels in the selected KO clones were confirmed by Western blot and flow cytometry (see below). sgRNA#1-edited Fn14-KO#1 clones for both GL261 and CT-2A cell lines were used for all *in vivo* studies.

Analysis of surface Fn14 expression in glioma cells by flow cytometry

Single-cell suspensions of Fn14-positive and Fn14-KO glioma cells were stained with Zombie Aqua fixable viability dye at room temperature in the dark for 30 mins. Following PBS washes, cells were pre-incubated with Trustain FcX (anti-mouse CD16/32) antibody (Biolegend, 101301) for 10 mins on ice before immuno-labeling with either APC-conjugated anti-human/mouse CD266 (Fn14, TWEAK R) antibody (Biolegend, 314107) or Mouse IgG2b, κ isotype control antibody (Biolegend, 402206) for 30 mins on ice. Cells were washed with fluorescence-activated cell sorting (FACS) staining buffer and fixed with 4% paraformaldehyde for 10 mins at room temperature. Samples were acquired using the BD LSR II flow cytometer, and data were analyzed using FlowJo software (FlowJo, Ashland, Oregon).

Western blot

Cells were lysed using 1x RIPA buffer (Sigma, R0278) supplemented with Halt protease and phosphatase inhibitor cocktail (Thermo Fisher Scientific, 1861281). For preparing tumor tissue lysates, brains harvested from tumor-bearing mice were homogenized using a probe sonicator in ice-cold 1x RIPA buffer (Sigma, R0278) containing protease and phosphatase inhibitors (Thermo Fisher Scientific, 1861281). Samples were centrifuged at 14,000 rpm for 10 mins at 4°C to pellet debris and the supernatant was collected. Protein concentration for all samples was determined using the BCA protein assay (Thermo Fisher Scientific, 23227). Equal amounts of protein were loaded per lane and resolved by SDS-PAGE using Nu-PAGE 12% gels (Thermo Fisher Scientific, NP0342), followed by transfer to PVDF membranes (Invitrogen, IB24002) using the iBlot2 transfer system. Membranes were then blocked in either 5% non-fat dry milk or bovine serum albumin (BSA) (for phosphorylated proteins) in 1x Tris-buffered saline with 0.1% Tween (TBS-T) (Cell Signaling Technology, 9997) and incubated overnight at 4°C with primary antibodies specified in Supplementary Table 1. Membranes were then incubated with appropriate horseradish peroxidase (HRP)-conjugated secondary antibody. HRP activity was detected using SuperSignal West Femto Maximum Sensitivity Substrate (Thermo Scientific, 34095). Images were acquired on the iBright Imaging System (Thermo Fisher Scientific). Densitometry was performed using ImageJ^1^. For protein abundance calculations, samples were first normalized to respective loading control and expressed as fold change compared to Fn14-positive controls. Raw uncropped images for all western blots are presented in Supplementary Figures 13-15.

Hematological profiling of Fn14-wildtype (WT) and -knockout (KO) mice:

Healthy Fn14-KO (Fn14^-/-^) mice and their WT littermate controls (n=5) were anesthetized with 4% chloral hydrate (400 mg/kg intraperitoneally), and blood was obtained via a transcardial puncture for cell count with differential analysis. All animal procedures were conducted following the guidelines of the Guide for the Care and Use of Laboratory Animals, with the approval of the Institutional Animal Care & Use Committee (IACUC) of Emory University, Atlanta GA.

Intracranial implantation of glioma cells

Mice were anesthetized using continuous delivery of isoflurane (>1-3%) via a nose cone and secured to a stereotaxic frame. Bupivacaine (2 mg/kg) was administered at the injection site before surgery. A handheld microdrill was used to create a burr hole in the right frontal lobe, approximately 2 mm lateral to the midline and 1 mm anterior to the coronal suture. Using a 26-gauge Hamilton syringe secured to the stereotaxic frame, glioma cells (CT-2A Fn14-positive or Fn14-KO cells, 1 x 10^5^ cells in 2 µl; GL261 Fn14-positive or Fn14-KO cells, 2 x 10^5^ cells in 2 µl) were injected 3 mm below the dura at 0.5 µl/min. Carprofen (4-5 mg/kg) was subcutaneously pre- and post-surgery. Mice were monitored daily for up to seven days for signs of pain, distress, or infection at the surgical site, followed by routine examinations for neurological dysfunction or distress. Body weights were recorded three times a week. Animals were euthanized if they lost more than 20% of their starting body weight or met other pre-determined criteria for alternative endpoints. All animal procedures were approved by the University of Maryland Institutional Animal Care and Use Committee (IACUC) and the Office of Animal Welfare Assurance (OAWA).

Bioluminescence imaging (BLI)

Mice were anesthetized in an induction chamber with continuous delivery of 2.5% isoflurane/100% oxygen at a flow rate of 1 L/min. D-luciferin (Thermo Scientific, 88293) dissolved in sterile phosphate-buffered saline was administered intraperitoneally at 150 mg/kg. After 10 mins, mice were moved to a Xenogen IVIS system (Caliper Life Sciences, Hopkinton, MA) maintained at 2.5% isoflurane and imaged for tumor bioluminescence. Photons emitted from live mice were acquired as photons/s/cm2/steradian (p/s/cm2/sr). Tumor burden was estimated by calculating the total photon flux (photons/sec) within identical regions of interest drawn around the brain using Living Image software (PerkinElmer, MA).

Immunohistochemistry

Briefly, tissue sections were deparaffinized, and rehydrated. Antigen retrieval was performed using proteinase K solution (Goldbio). Slides were then incubated in 3% hydrogen peroxide (H_2_O_2_) for 15 mins, followed by blocking with 2% goat serum in Tris-HCl for 15 mins. Tissue sections were incubated overnight with anti-F4/80 primary antibody (1:200, Biorad, MCA497GA) at 4°C. The next day, biotinylated secondary (1:500, Invitrogen, 31830,) was applied. Streptavidin ABC-kit (Cat#, Vector Laboratories) and DAB substrate kit (Sigma, D4293) were used for detection. The tissues were counterstained with hematoxylin QS (Vector Laboratories, H-3404) and mounted with Permount after dehydration. CD3 staining was performed by the University of Maryland Pathology Biorepository Shared Services (PBSS) core using the Dako EnVision FLEX + detection system (DAKO, Carpinteria, CA). FFPE tissue sections were deparaffinized with Dako PT link followed by heat-induced epitope retrieval using Dako Target Retrieval Solution, High pH, for 20 mins. Endogenous peroxidase activity was blocked with DAKO Peroxidase-Blocking Reagent for 5 mins before incubation with FLEX Polyclonal Rabbit Anti-Human CD3, Ready-to-Use (Link) (DAKO, Carpinteria, CA) for 20 mins at room temperature followed by DAKO Anti-rabbit HRP Detection Reagent for 20 mins. Finally, the sections were incubated for 10 mins with DAKO diaminobenzene (DAB), counterstained with Dako FLEX hematoxylin, rinsed, and mounted in Cytoseal XYL (Thermo Scientific, Waltham, MA). All slides were scanned using Aperio Scanscope at 20X magnification. Positive staining for F4/80 was quantified using the positive pixel count macro from Aperio Imagescope software. Positive staining for CD3 was evaluated by a board-certified neuropathologist in a blinded fashion.

Immunofluorescence imaging

For human GBM samples, tissue sections were blocked for 1 hr in 2% donkey serum with 0.2% Triton X-100 and labeled overnight at 4°C with primary antibodies against Iba1 (1:100, Abcam, ab5076) and Fn14 (Abcam, 109365,). Alexa Fluor 500- or fluorescein isothiocyanate-conjugated secondary antibodies were applied, and tissues were cover slipped with ProLong Gold antifade reagent (P36930, Thermo Fisher Scientific Inc.). For mouse brain tumor samples, coronal FFPE sections were obtained from the center of the tumor's anterior-posterior extent. Tissue sections were deparaffinized, rehydrated, and heat-based antigen retrieval (Antigen Unmasking Solution, Citric Acid, Vector Labs). Sections were then blocked with 5% BSA, 0.1% Tween in PBS for 1 hr at room temperature and labeled overnight at 4°C with anti-TWEAKR/Fn14 (Abcam, 109365) and AIF-1/Iba1 (Biotechne, NB100-1028) primary antibodies. The next day fluorescently tagged secondary antibodies were applied for 1 hr at room temperature. The tissues were cover slipped with Fluoroshield containing DAPI (Abcam). Specific labeling was confirmed by the omission of primary antibody on tumor tissue. Immunolabeled tissues were visualized on the Nikon W-1 spinning disk confocal microscope (Nikon, Tokyo, Japan).

Flow cytometric analysis of brain tumors:

Tumor-bearing brain hemispheres were isolated and mechanically dissociated between two frosted microscopy slides. Tissues were digested in 1 mg/mL collagenase A (Roche) in a solution of 2% heat-inactivated FBS (Gibco) and RPMI-1640 (Gibco) for 20 mins at 37ºC/5% CO2. Suspensions were filtered and washed. To separate myelin, samples were treated with a 90% Percoll solution (Cytiva) and centrifuged at 4°C for 15 mins at 500 x g (acceleration 9, deceleration 5). Cells in the pellet were retained for experiments and suspended in MACS buffer (0.5% BSA (Fisher), 2 mM EDTA (Fisher) in PBS). Cell suspensions from tissue were filtered and subjected to RBC lysis (ACK Lysing Buffer) followed by incubation with Fc block (BioLegend). Next, cells were incubated with fluorescently tagged antibodies (see Supplementary Table1) against surface antigens for 20 mins on ice. Following washes with MACS buffer, samples were acquired on a Cytek Auora 5 flow cytometer. Data were analyzed using SpectroFlo software (Cytek Biosciences). The gating strategy is illustrated in Supplementary Figure S10.

Single-cell RNA-sequencing data analysis

Single-cell RNA sequencing data for human GBM tumors from the GSE84465 dataset^2^ were accessed at <http://gbmseq.org> to visualize cell type-specific Fn14 *(TNFRSF12A)* gene expression in the human GBM TME. Data from all samples in this dataset was used to generate the plot in Figure 1A.

Gene Set Enrichment Analysis (GSEA)

RNA-sequencing data for 122 IDH wild-type samples from a multiregional high-grade glioma biopsy dataset (syn52256654)^5^ were downloaded, normalized and batch corrected as described previously^5^. GBMDeconvoluteR (https://gbmdeconvoluter.leeds.ac.uk/Home) was used to estimate tumor purity for all samples. Differential gene expression analysis was performed using the DESeq2 package (v.1.42.1)^6^ with the ‘design’ parameter to adjust for tumor purity effects. The Wald test was used to compare gene expression between “High” vs. “Low” Fn14 expression groups and the resulting genes were ordered by the DESeq2 ‘stat’ metric for downstream enrichment analysis. The full list of ranked genes was used as input for gene set enrichment analysis (GSEA) using the clusterProfiler R package (v.4.10.1)^7^. Curated gene sets were obtained from MSigDB (C5, GO: Biological Processes)^8,9^ via the msigdbr package (v.10.0.1)^10^ and were further filtered to include only gene sets related to the parent GO term GO:0002376 “immune system process”, gathered from https://www.ebi.ac.uk/QuickGO. Enriched biological process terms with a Benjamini-Hochberg adjusted p-value < 0.05 were summarized and visualized using the rrvgo package (v.1.14.2). Semantic similarity among enriched terms was computed using the “Rel” method in the Biological Process ontology. Terms were clustered and reduced using a similarity threshold of 0.5, with representative parent terms retained. Bar plots of top immune pathways were generated using ggplot2 (v.3.5.2). TreemapPlot and scatterPlot() were used to visualize pathways in Supplementary Figure 12.

Single sample Gene Set Enrichment Analysis (ssGSEA)

Raw data from syn52256654^5^ dataset were normalized to transcripts per million (TPM) with the method established in Varn et al.^11^ using ENSEMBL IDs for all genes. A negligible number of genes (6%) with no available gene length information were dropped. The batch correction was performed using Combat_seq with the sva package (v.3.54.0) in R^12,13^. ENSEMBL IDs were mapped to gene names using the org.Hs.eg.db package (v.3.20.0) in R^14^. ssGSEA v.10.1.0 from GenePattern^15^was run on batch corrected TPMs to calculate enrichment scores for each patient sample using the same reference gene set as above (with GSEA). To ensure robustness, only gene sets with a minimum of 15 genes in the dataset were used. All IDH-WT samples were classified into Fn14-high and Fn14-low groups based on the top- and bottom-quartile Fn14 gene expression values from batch-corrected TPMs. Mann-Whitney U test with False Discovery Rate correction was used to compare pathway enrichment scores between Fn14-high and Fn14-low groups and determine statistically significant differences (adjusted p-value < 0.05). Redundant pathways were removed using the rrvgo package^16^ (v.1.18.0) in R based on REVIGO^17^. Semantic similarity among enriched terms was computed using the “Rel” method. Terms were clustered and reduced using a similarity threshold of 0.5, with representative parent terms retained. Visualization and associated preprocessing of results were performed using the ComplexHeatmap (v.2.22.0)^18,19^, ggplot2 (v.3.5.2)^20^, reshape2 (v.1.4.4)^21^, dplyr (v.1.1.4)^22^, cowplot (v.1.1.3)^23^, and circlize (v.0.4.16)^24^ packages in R.

CIBERSORTx analysis:

RNA-sequencing data from syn52256654^5^ dataset was downloaded and processed as described above. Samples were classified as Fn14-high and Fn14-low based on top and bottom quartile *TNFRSF12A* gene expression values, respectively. Immune cell fractions in the tumor microenvironment were inferred using CIBERSORTx web platform (https://cibersortx.stanford.edu/index.php) and the provided LM22 cell signature file. Samples were analyzed in ‘absolute’ mode with batch correction enabled over 500 permutations. A Wilcoxon rank sum test was performed for each cell type (*p < 0.05, **p < 0.01, ***p < 0.001).

Correlation analysis for Fn14 and immune marker genes:

Gene expression data were extracted for Fn14 (*TNFRSF12A*) and immune response related genes (*Pdcd1, Cd274, Pdcd1lg2, Lag3, Tigit, Ccl2, Il6, Tgfb1*) and z-score normalized across samples. Linear mixed-effects models with random intercepts for patient ID were used to account for patient-level variability in the association between Fn14 and each gene using the lme4 and lmerTest R packages (v.1.1-37, v.3.1-3).

Kaplan-Meier survival analysis of GBM immunotherapy datasets

For survival analysis of GBM immunotherapy outcomes, RNA-sequencing data for GBM (IDH-WT only) patients who received adjuvant pembrolizumab (anti-PD1) therapy was downloaded from the GSE121810 dataset^8^. Survival data was obtained from McFaline-Figueroa J, et al^25^. The DESeq2 package (v.1.46.0)^6^ in R was used to normalize raw counts^9^. Median gene expression was used to stratify patients into Fn14-high and -low groups. Kaplan-Meier survival curves were generated using overall survival data, and Log-rank (Mantel-Cox) test was used to determine statistical significance using GraphPad Prism software.

Statistical analysis:

Data are presented as the mean ± SEM unless specified otherwise. Kaplan-Meier survival curves were compared using the Log-rank test, with median survival times assessed using Mann-Whitney U test. Unpaired Student’s t-test or Wilcoxon rank-sum test was used to compare data from two groups unless specified otherwise. Mixed-effect model was used to analyze paired data. One-way ANOVA, with appropriate post hoc test was used for multiple comparisons of normally distributed data. Mann Whitney U test (two groups) or Kruskal-Wallis test (multiple groups) with appropriate post hoc test was used for comparing non-normal distributions. A p<0.05 was considered statistically significant unless indicated otherwise. Statistical analyses were performed, and graphs were created using GraphPad Prism 10.0 or R. Additional details can be found in the respective figure legends.

**Supplementary Figures:**

**
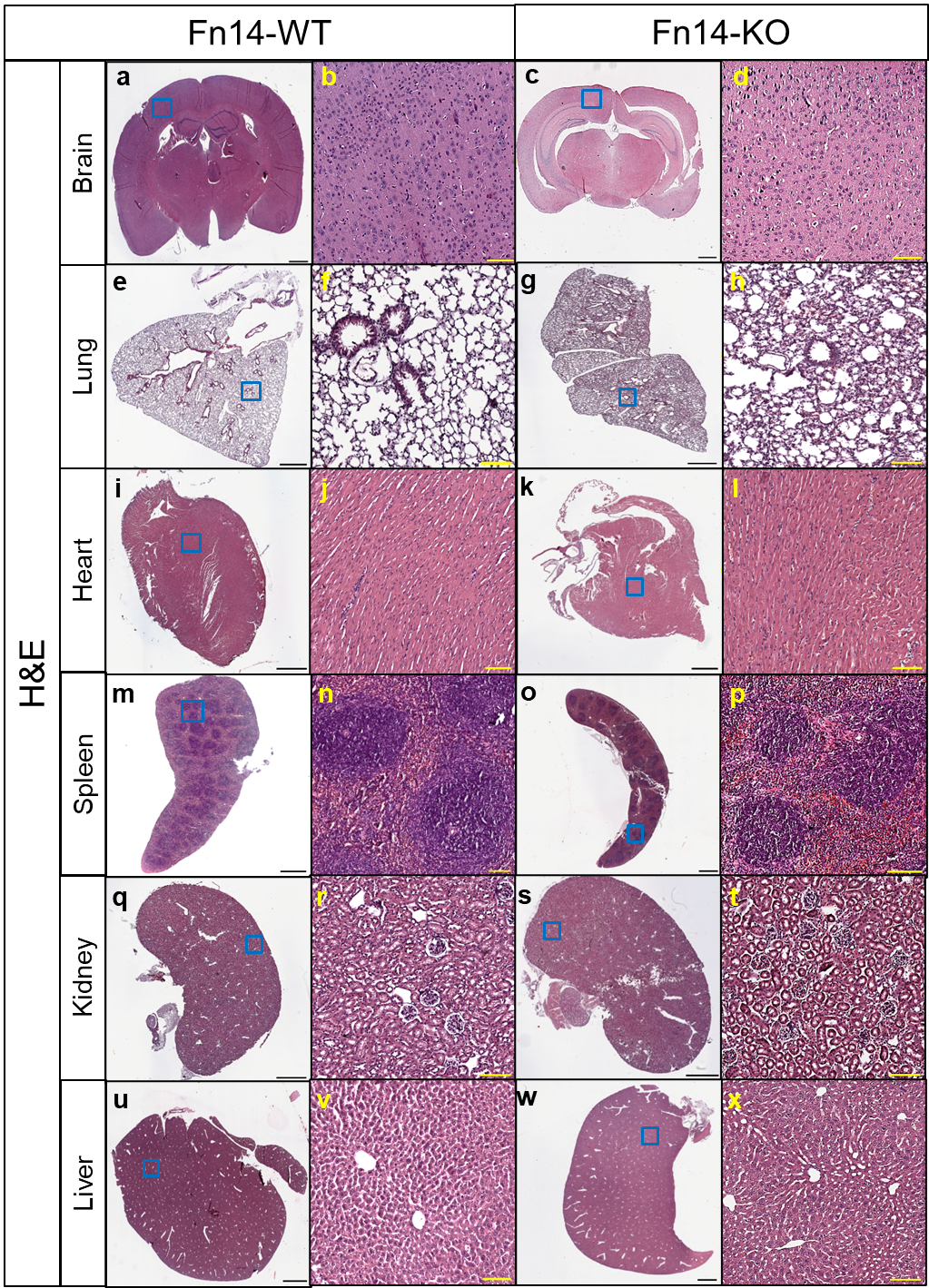
**

**Supplementary Figure 1. Histological analysis of healthy tissues from Fn14-WT and -KO mice.** Brain, lung, heart, spleen, kidneys and liver were harvested from healthy Fn14-WT and -KO mice; paraffin embedded, and Hematoxylin and Eosin (H&E) staining was performed. Histological features of various tissues were examined in a blinded fashion. Scale bars: a, c, e, g, i, k, m, o, q, s = 1mm; u, w = 2 mm; b, d, f, h, j, l, n, p, r, t = 100 μm.


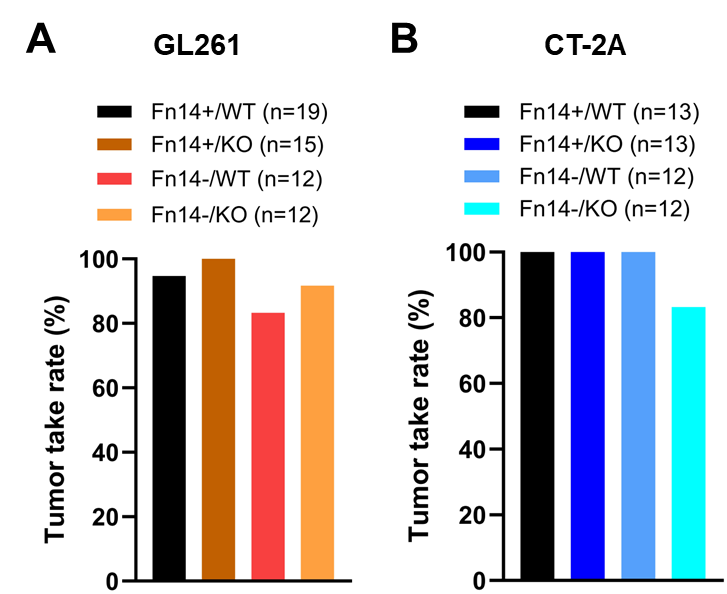


**Supplementary Figure 2. Analysis of tumor take rates for GL261 and CT-2A glioma models.** Fn14-WT and Fn14-KO mice were orthotopically injected with GL261 or CT-2A Fn14-positive or Fn14-KO glioma cells. One-week post-injection, bioluminescence imaging (BLI) was performed to confirm the presence of the tumor. Tumor take rate was calculated as follows: [(number of mice with positive BLI signal one-week post-injection)/(total number of mice injected)] x 100. Tumor take rates for **(A)** GL261 (Fn14+/WT, black, n=19; Fn14+/KO, brown, n=15; Fn14-/WT, red, n=12; Fn14-/KO, gold, n=12) and **(B)** CT-2A (Fn14+/WT, black, n=13; Fn14+/KO, deep blue, n=13; Fn14-/WT, light blue, n=12; Fn14-/KO, cyan, n=12). Fischer’s exact test was used to compare tumor take rates. p<0.05 was considered statistically significant.


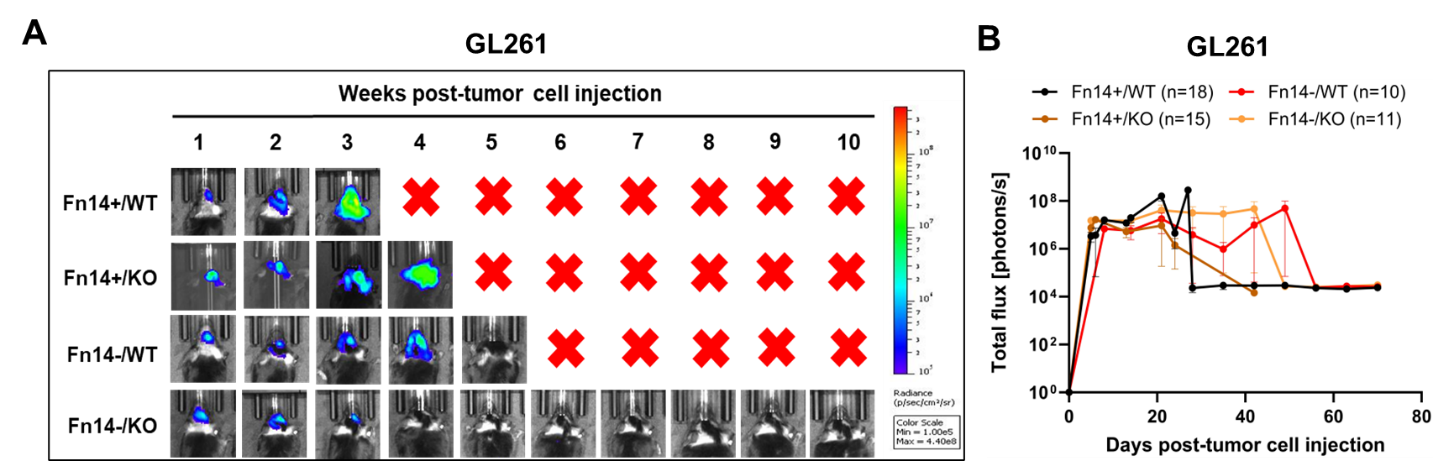


**Supplementary Figure 3. Assessment of in vivo tumor growth for Fn14-WT and Fn14-KO mice with Fn14-positive and Fn14-KO GL261 tumors. (A)** Representative images from bioluminescence imaging (BLI) of Fn14-WT and Fn14-KO mice orthotopically implanted with either Fn14-positive or Fn14-KO GL261 glioma cells. **(B)** Quantification of BLI signal from (A) (Fn14+/WT, black, n=18; Fn14+/KO, brown, n=15; Fn14-/WT, red, n=10; Fn14-/KO, gold, n=11). Data are presented as mean ± SEM.


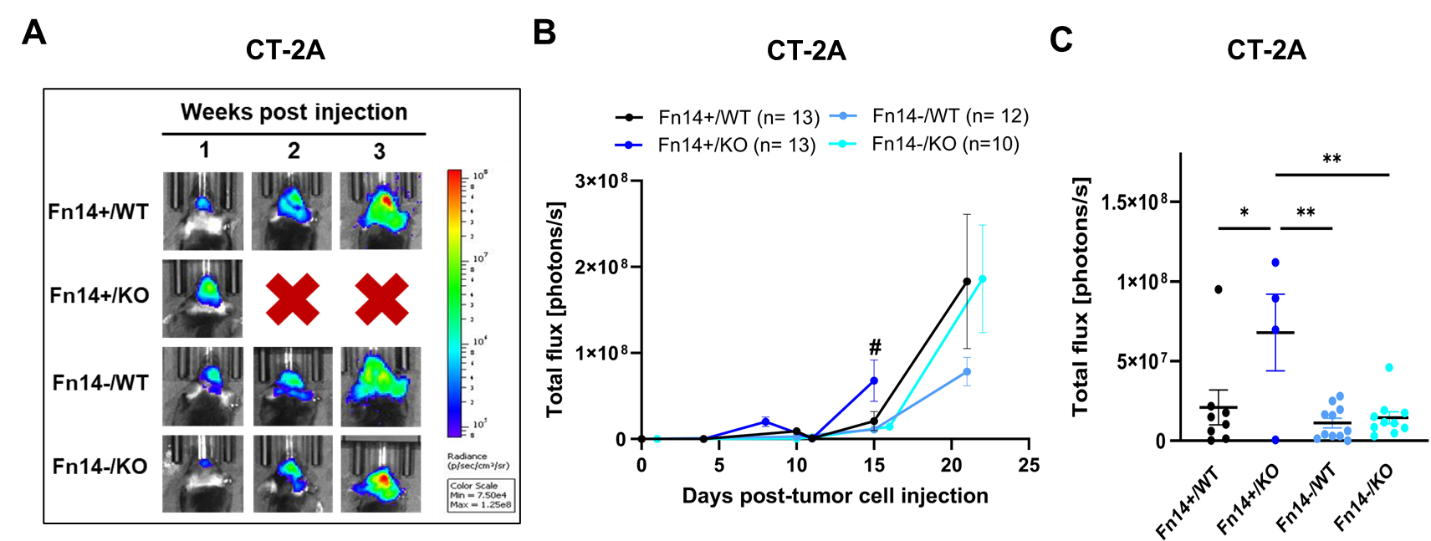


**Supplementary Figure 4. Assessment of in vivo tumor growth for Fn14-WT and Fn14-KO mice with Fn14-positive and Fn14-KO CT-2A tumors. (A)** Representative images from bioluminescence imaging (BLI) of Fn14-WT and Fn14-KO mice orthotopically implanted with either Fn14-positive or Fn14-KO CT-2A glioma cells. **(B)** Quantification of BLI signal from (A). (Fn14+/WT, black, n=13; Fn14+/KO, cyan, n=13; Fn14-/WT, light blue, n=12; Fn14-/KO, deep blue, n=10) **(C)** Comparison of total BLI signal at day 15 post-tumor cell injection. (Fn14+/WT, black, n=8; Fn14+/KO, cyan, n=4; Fn14-/WT, light blue, n=11; Fn14-/KO, deep blue, n=10). One-way ANOVA with Tukey’s test for multiple comparisons was used to determine statistical significance at p<0.05 (*p<0.05, **p<0.01). Data are presented as mean ± SEM.


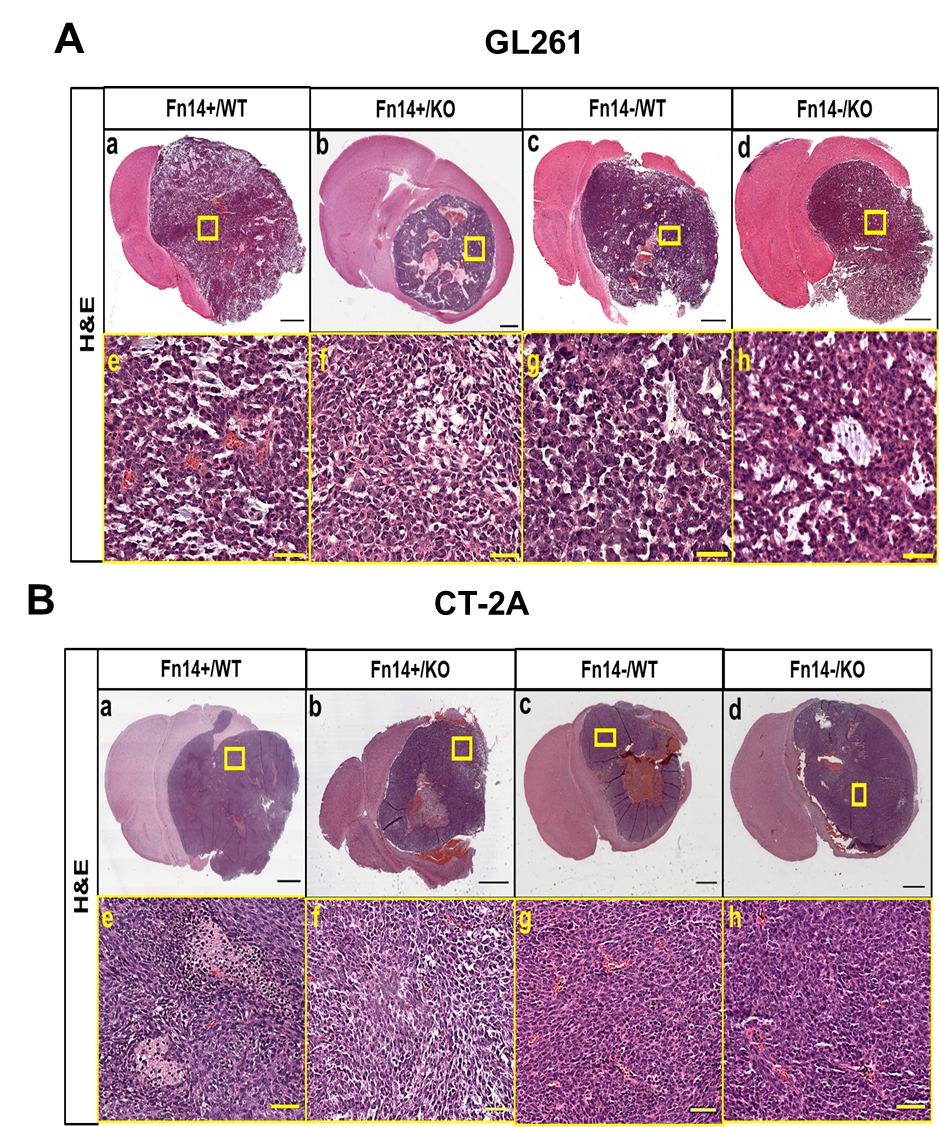


**Supplementary Figure 5. Histological analyses of Fn14-positive and Fn14-KO brain tumor samples harvested from Fn14-WT and Fn14-KO mice.** At the survival endpoint, animals were euthanized, brain tissue was collected, paraffin embedded, and Hematoxylin and eosin (H&E) staining was performed. Slides were blinded and evaluated by a board-certified neuropathologist for histological abnormalities. Yellow squares show regions from the tumor core. Representative H&E images for **(A)** GL261 and **(B)** CT-2A tumors. Scale bars: a-d = 1mm; e-h = 50 μm.


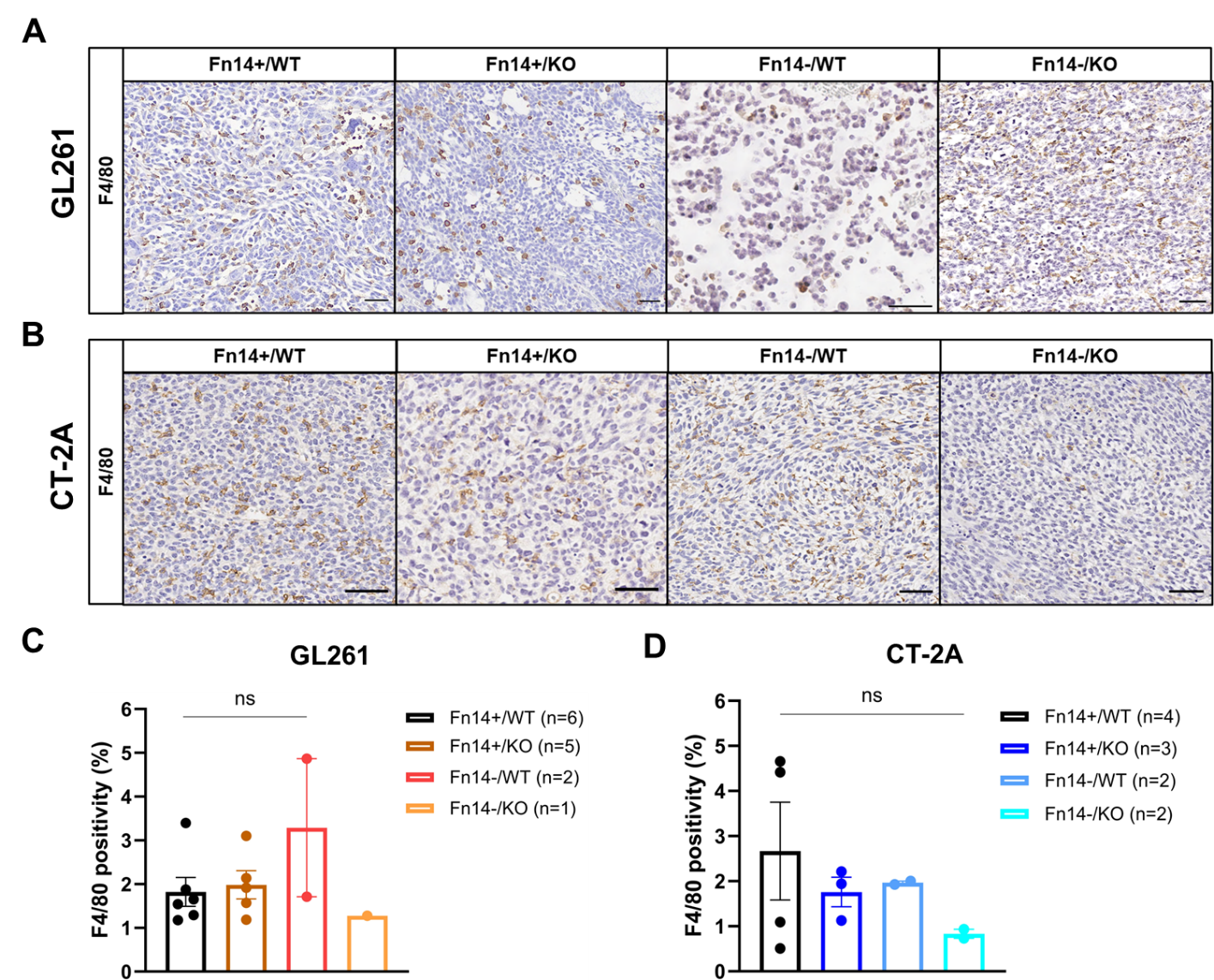


**Supplementary Figure 6. Immunohistochemical analyses of macrophage infiltration in Fn14-positive and Fn14-KO tumors harvested from Fn14-WT and Fn14-KO mice.** At the survival endpoint, animals were euthanized, brain tissue was collected, paraffin was embedded, and immunohistochemistry for macrophage marker F4/80 was performed. F4/80 positive staining was quantified using the positive pixel count algorithm from Aperio Imagescope. IHC staining and quantification of F4/80 positive cells in **(A)** GL261 tumors (Fn14+/WT, black, n=6; Fn14+/KO, brown, n=3; Fn14-/WT, red, n=2; Fn14-/KO, gold, n=1) and **(B)** CT-2A tumors (Fn14+/WT, black, n=4; Fn14+/KO, deep blue, n=3; Fn14-/WT, light blue, n=2; Fn14-/KO, cyan, n=2). Scale bars = 50 μm Kruskal-Wallis test with Dunn’s test for multiple comparisons was used to determine statistical significance at p<0.05.


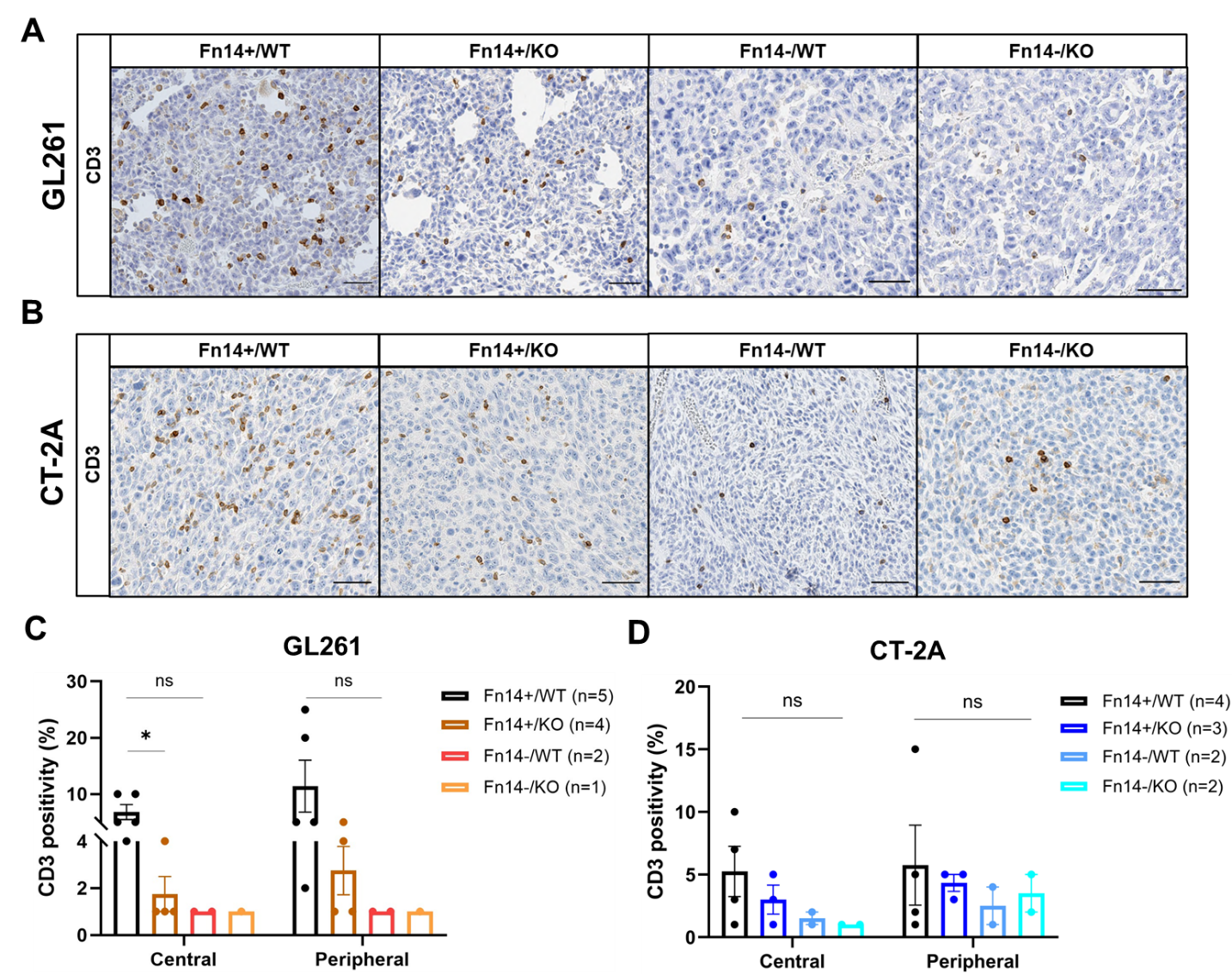


**Supplementary Figure 7. Immunohistochemical analyses of T-cell infiltration in Fn14-positive and Fn14-KO tumors from Fn14-WT and Fn14-KO mice.** At the survival endpoint, animals were euthanized, brain tissue was collected, paraffin was embedded, and immunohistochemistry for pan T-cell marker CD3 was performed. Slides were blinded and scored for central and peripheral CD3+ cells by a board-certified neuropathologist. IHC staining and quantification of CD3+ cells in **(A)** GL261 tumors (Fn14+/WT, black, n=5; Fn14+/KO, brown, n=4; Fn14-/WT, red, n=2; Fn14-/KO, gold, n=1) and **(B)** CT-2A tumors (Fn14+/WT, black, n=4; Fn14+/KO, deep blue, n=3; Fn14-/WT, light blue, n=2; Fn14-/KO, cyan, n=2). Scale bars = 50 μm. Scale bars = 50 μm. Kruskal-Wallis test with Dunn’s test for multiple comparisons was used to determine statistical significance at p<0.05 (*p<0.05).


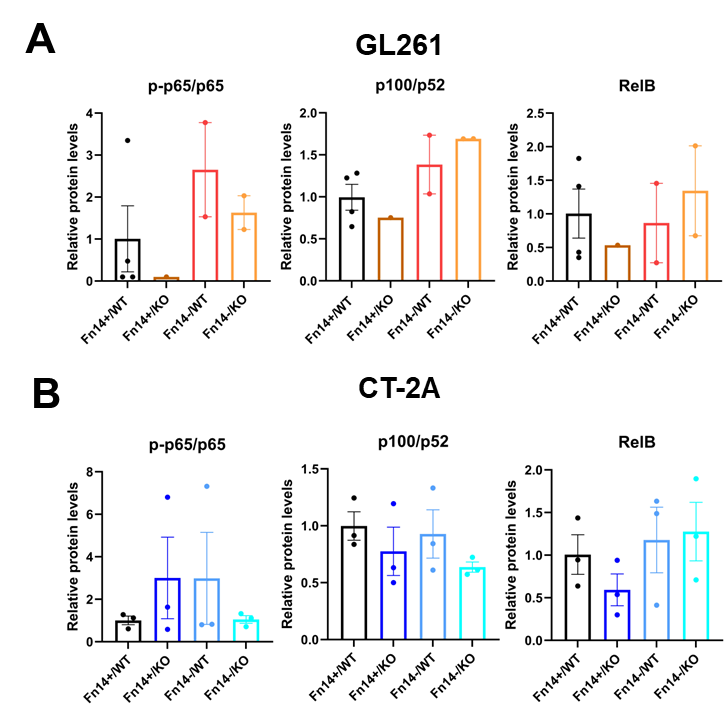


**Supplementary Figure 8. Densitometry analysis of Western blot data for NF-κB signaling pathway proteins in Fn14-positive and Fn14-KO tumor-host pairs.** At the survival endpoint, brain tissues were collected, protein lysates prepared, and Western blot analysis was conducted to detect expression of canonical (phospho-p65, p65) and non-canonical (NF-κB2, RelB) NF-κB pathway proteins (see Figure 3E, F). Densitometry was performed to quantify protein expression. All protein levels were first normalized to GAPDH levels and represented as fold change relative to Fn14+/WT samples. Lanes represent samples collected from independent animals. Results for **(A)** GL261 (Fn14+/WT, black, N=4; Fn14+/KO, brown, N=1; Fn14-/WT, red, N=2; Fn14-/KO, N=2) and **(B)** CT-2A samples. (N=3 each for Fn14+/WT, black; Fn14+/KO, deep blue; Fn14-/WT, light blue; Fn14-/KO, cyan). Kruskal-Wallis test with Dunn’s test for multiple comparisons was used to determine statistical significance at p<0.05.


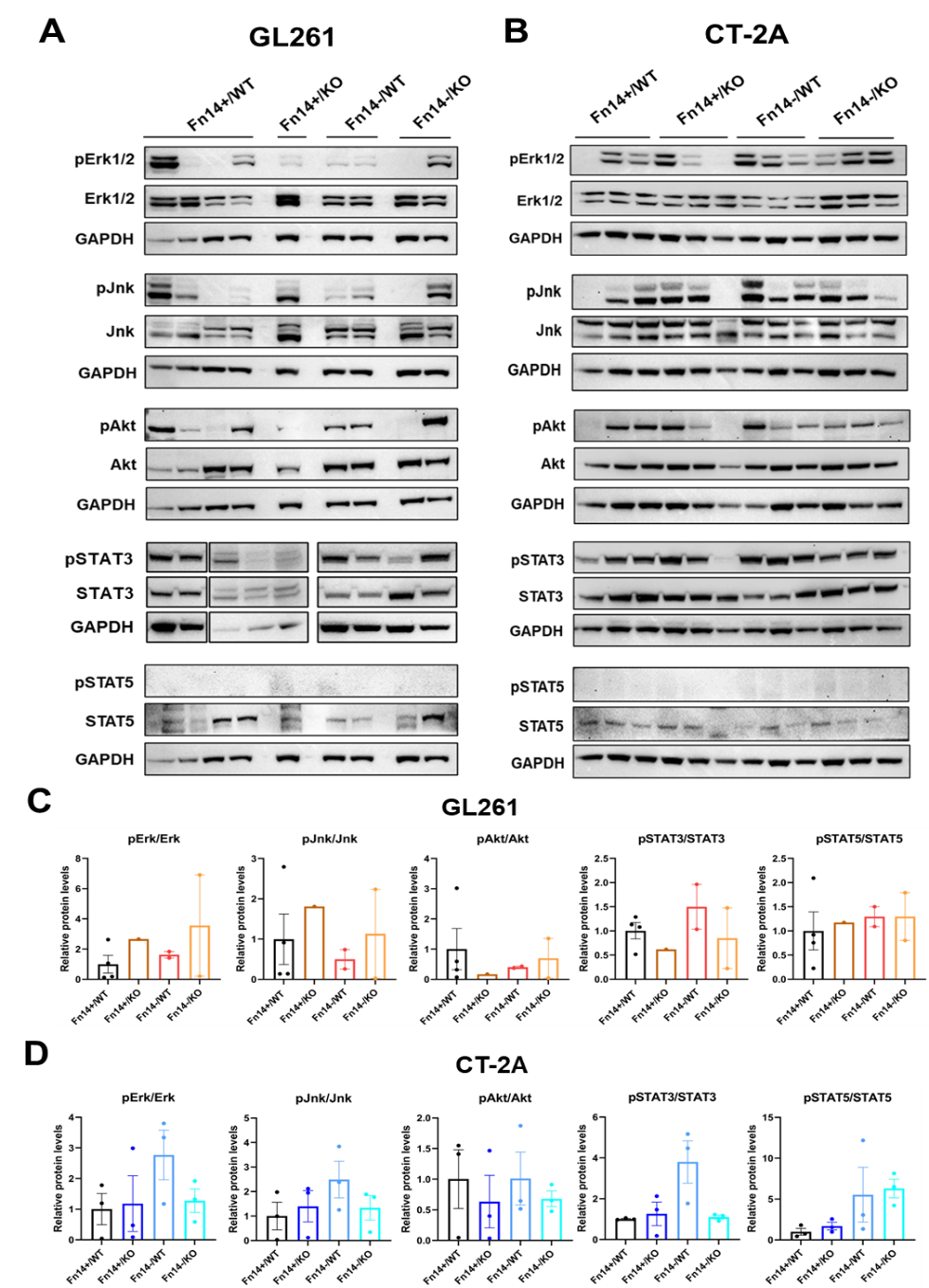


**Supplementary Figure 9. Western blot analysis of endpoint brain tumor samples for MAPK, Akt and JAK/STAT signaling pathways.** At the survival endpoint, brain tissues were collected, protein lysates were prepared, and expression levels of proteins involved in MAPK, Akt and JAK/STAT signaling pathways were assessed by Western blot. Densitometry was used to quantify protein expression. All values were first normalized to GAPDH levels and presented as fold change relative to Fn14+/WT samples. Lanes represent samples collected from independent animals. Western blot data for **(A)** GL261 and **(B)** CT-2A Fn14+/KO tumor-host pairings. **(C)** Densitometric analyses of data from (A). (Fn14+ cells/WT mice, black, N=4; Fn14+ cells/KO mice, brown, N=1; Fn14-KO cells/WT mice, red, N=2; Fn14-KO cells/KO mice, N=2). **(D)** Densitometric analyses of data from (B). (N=3 each for Fn14+ cells/WT mice, black; Fn14+ cells/KO mice, deep blue; Fn14-KO cells/WT mice, light blue; Fn14-KO cells/KO mice, cyan). Kruskal-Wallis test with Dunn’s test for multiple comparisons was used to determine statistical significance at p<0.05.


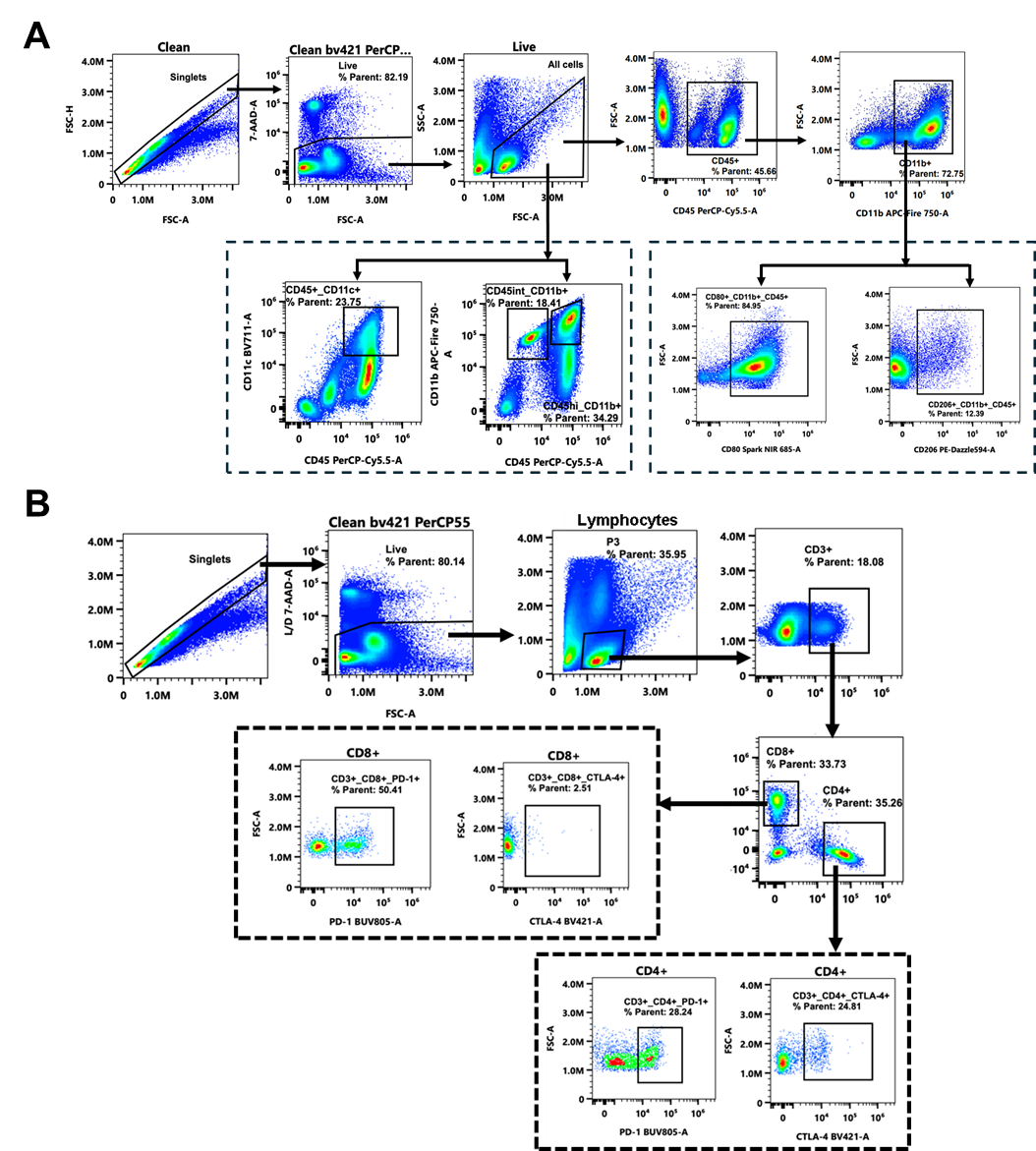


**Supplementary Figure 10. Gating strategy for identifying tumor-associated immune cell populations.** Representative gating strategy for **(A)** innate immune cells and **(B)** adaptive immune cells.


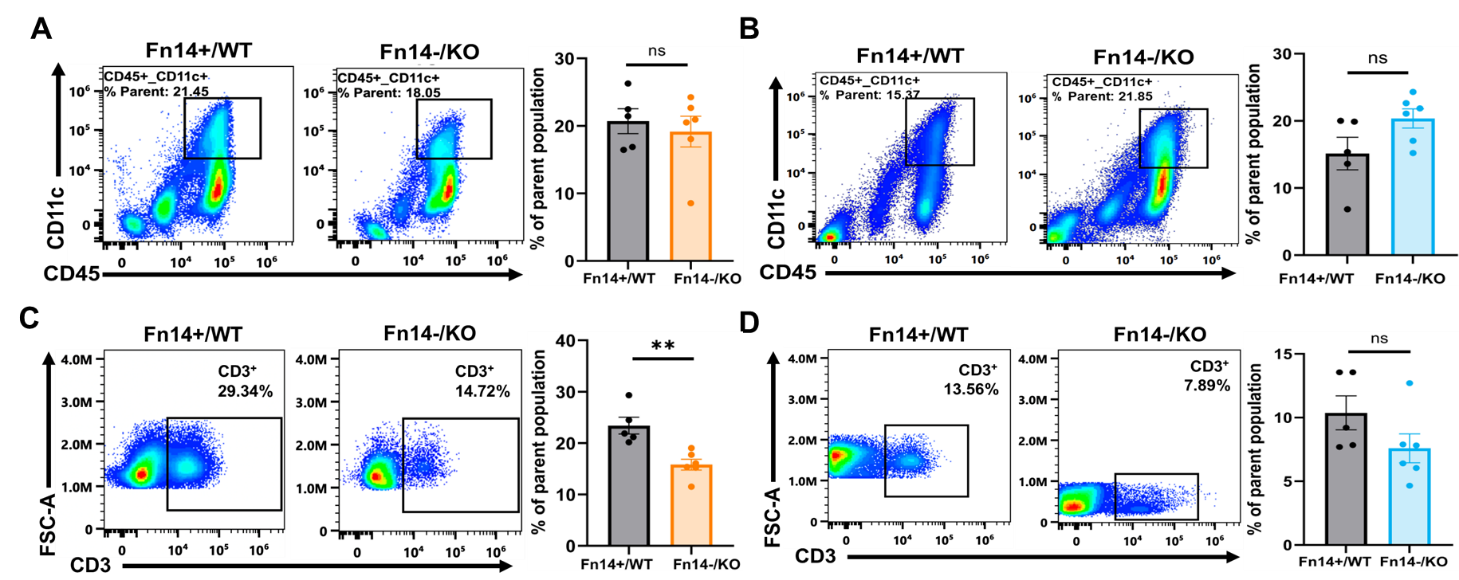


**Supplementary Figure 11. Relative frequency of dendritic cells and T-cells in Fn14+/WT and Fn14-KO/KO GL261 and CT-2A gliomas.** **(A-B)** Percentage of dendritic cells (CD45+ CD11c+) in **(A)** GL261 and **(B)** CT-2A Fn14+/WT and Fn14-KO/KO tumor-host pairs. **(C-D)** Percentage of CD3+ T-cells in **(C)** GL261 and **(D)** CT-2A Fn14+/WT and Fn14-KO/KO tumor-host pairs. Sample sizes: Fn14+ cells/WT mice, n=5 and Fn14-KO cells/KO mice, n=6 for both GL261 and CT-2A models. Unpaired Student’s t-test was used to determine statistical significance at p<0.05 (**p<0.01). Data are presented as mean ± SEM


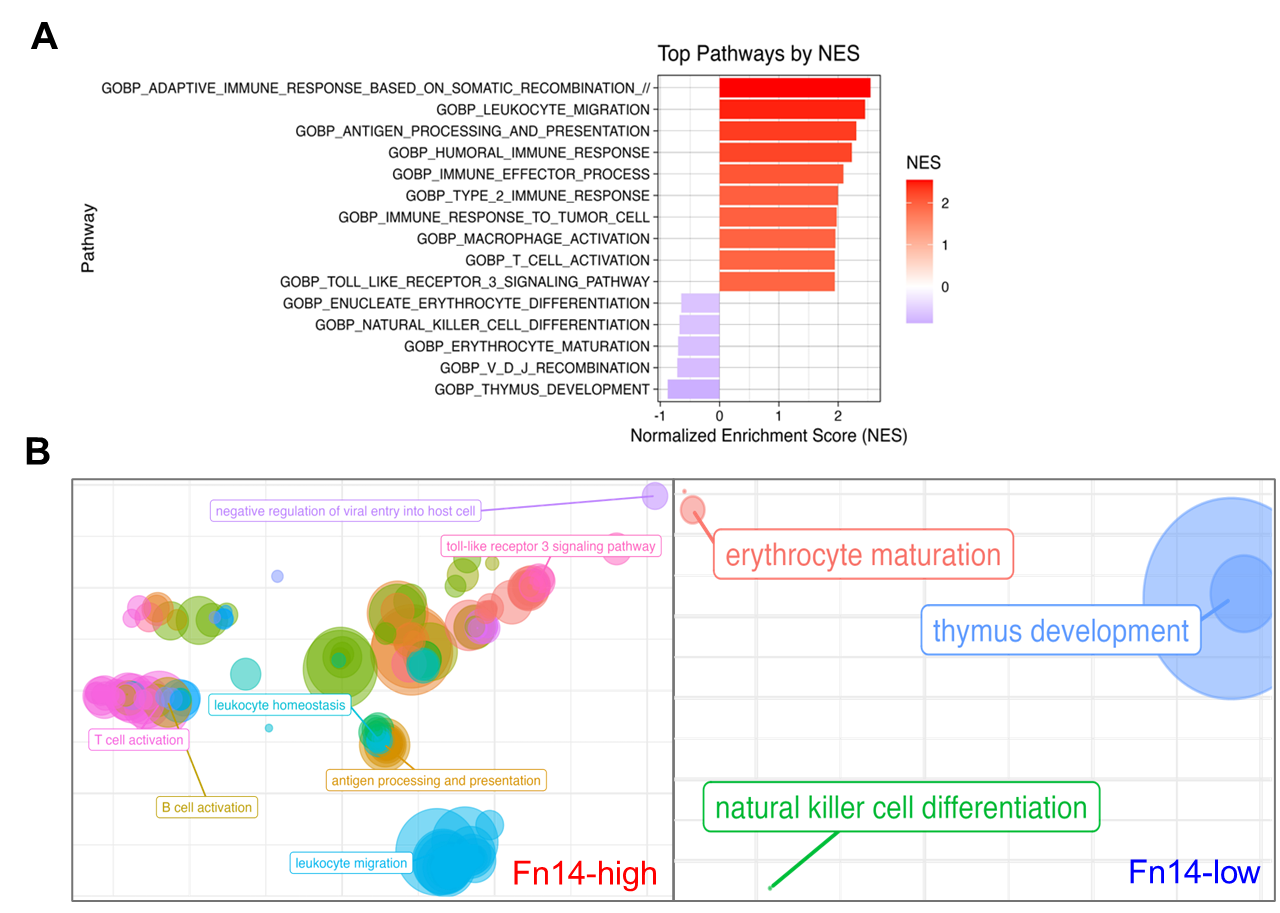


**Supplementary Figure 12. Gene Set Enrichment Analysis identifies differentially enriched immune process-related pathways in Fn14-high and Fn14-low human GBM tumors. (A)** Bar plot representing the top immune response associated biological processes enriched in Fn14-high (red, top quartile, n=31) (NES > 0) and Fn14-low (blue, bottom quartile, n=31) samples (NES < 0) following Gene Set Enrichment Analysis (GSEA) of IDH-WT human GBM tumors from the syn52256654 dataset. All Fn14-high terms have an adjusted p-value < 0.05. P-value filtering was not performed for Fn14-low terms due to weaker enrichment signal. **(B)** Bubble plots depicting rrvgo summarized Biological Process GO term enrichment for Fn14-high and Fn14-low samples.

**
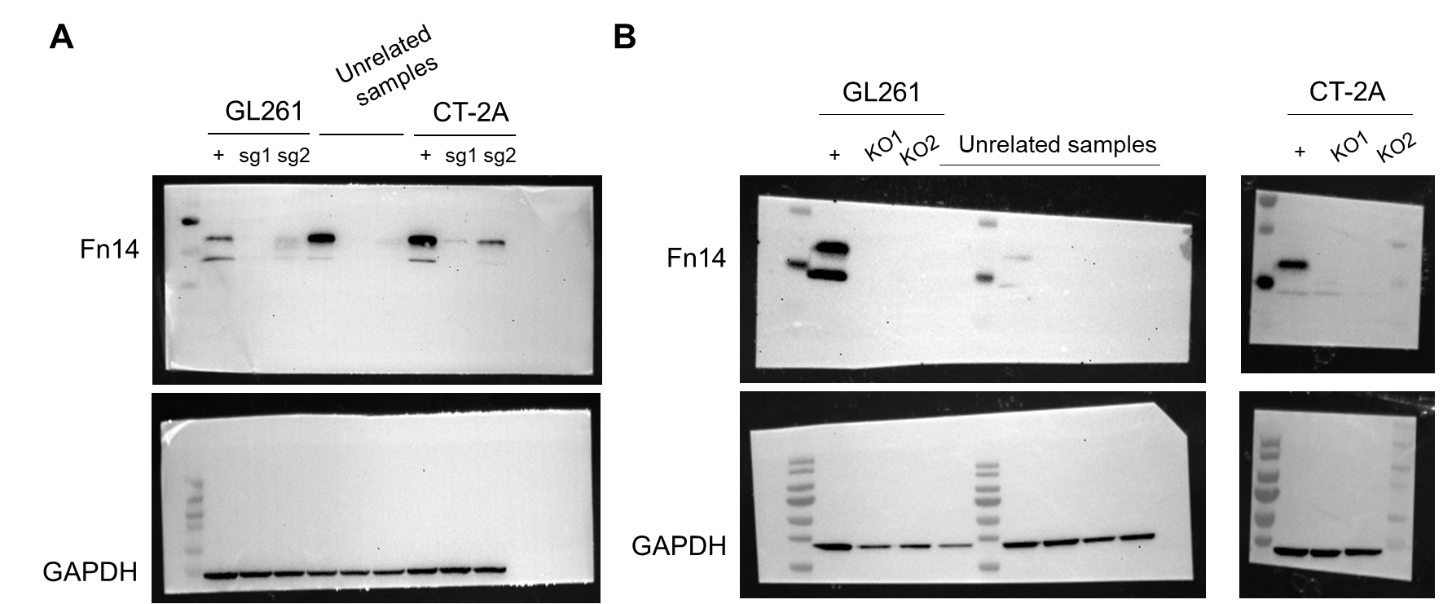
**

**Supplementary Figure 13.** Raw uncropped images for Western blot data shown in **(A)** Figure 2B and **(B)** Figure 2C.

**
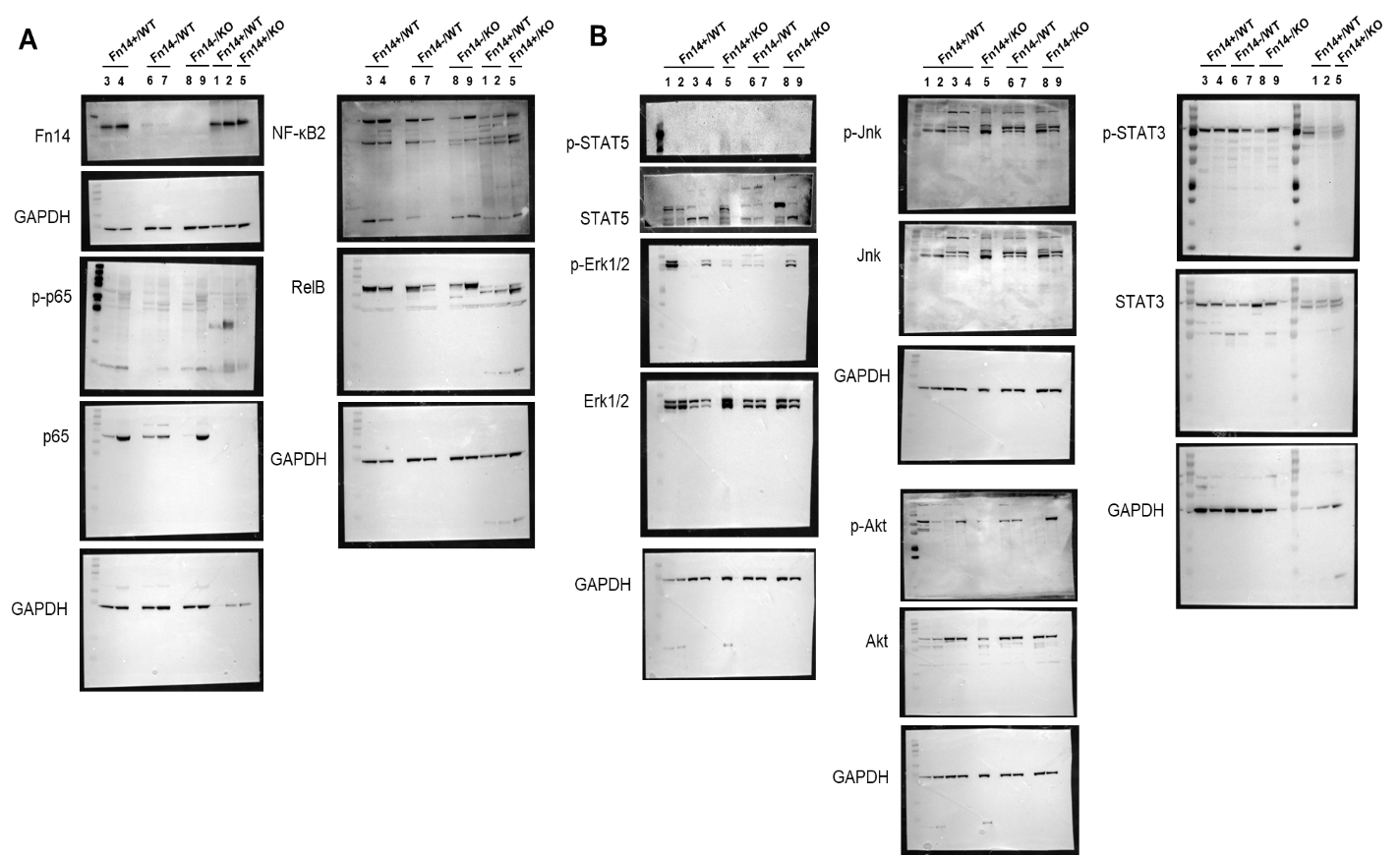
**

**Supplementary Figure 14.** Raw uncropped images for Western blot data presented in **(A)** Figure 3E and **(B)** Supplementary Figure 9A. The numbers correspond to the sample lanes and indicate the order in which samples appear in Figure 3E and Supplementary Figure 9A.

**
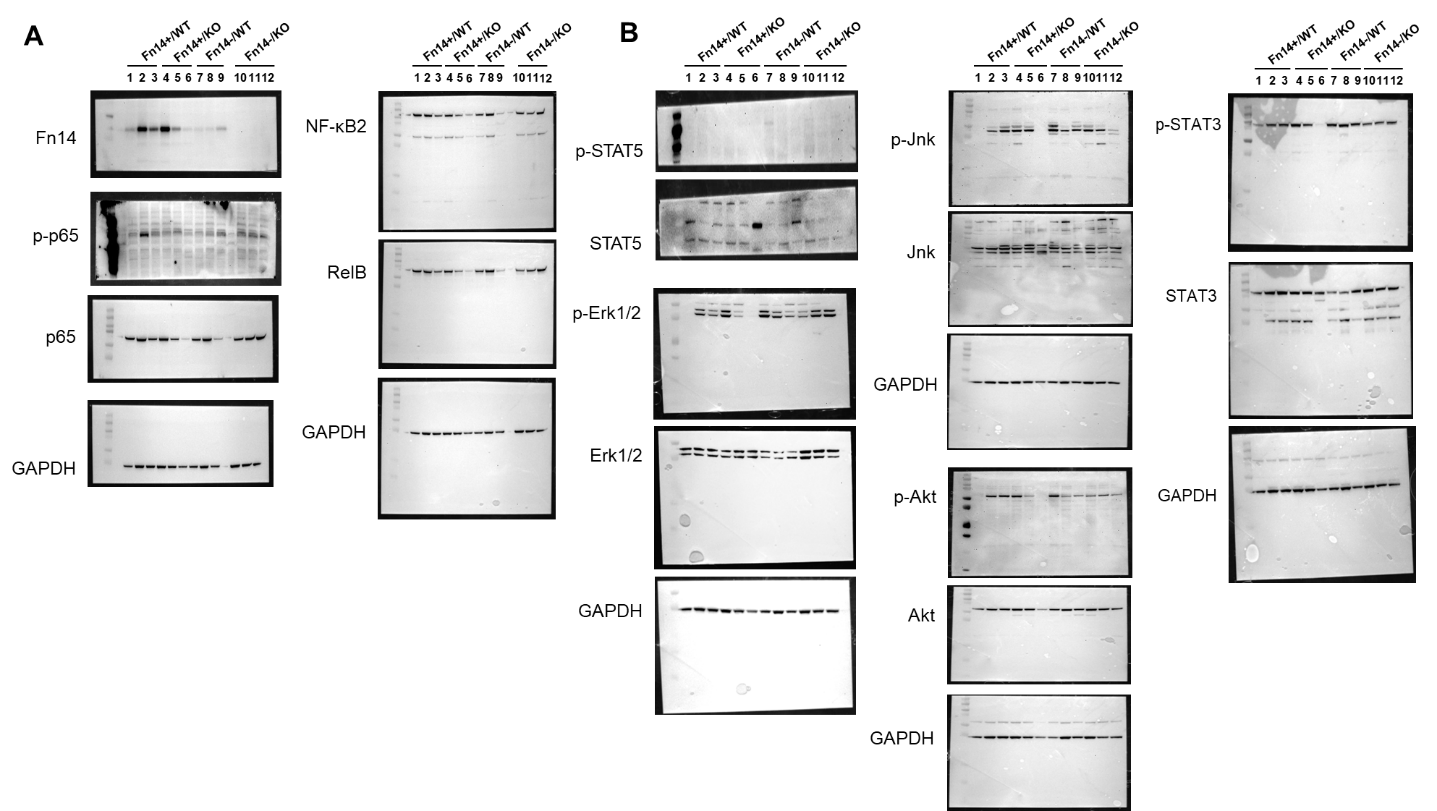
**

**Supplementary Figure 15.** Raw uncropped images for Western blot data presented in **(A)** Figure 3F and **(B)** Supplementary Figure 9B. The numbers correspond to the sample lanes and indicate the order in which samples appear in Figure 3F and Supplementary Figure 9B.

**Supplementary Tables:**

**Supplementary Table 1. List of antibodies for Western blot and flow cytometry**

| **REAGENT** | **VENDOR** | **CATALOG NO.** |
| --- | --- | --- |
| **Antibodies for Western blot:** | | |
| Fn14 | Abcam | 109365 |
| phospho-NF-κB p65 | Cell Signaling Technology | 3033 |
| NF-κB p65 | Cell Signaling Technology | 8242 |
| NF-κB2 (p100/p52) | Cell Signaling Technology | 4882 |
| RelB | Cell Signaling Technology | 4922 |
| phospho-Akt | Cell Signaling Technology | 9271 |
| Akt | Cell Signaling Technology | 9272 |
| phospho-Erk1/2 (p44/p42) | Cell Signaling Technology | 9101 |
| Erk 1/2 (p44/p42) | Cell Signaling Technology | 9102 |
| phospho-Jnk/SAPK | Cell Signaling Technology | 9251 |
| Jnk/SAPK | Cell Signaling Technology | 9252 |
| phospho-STAT3 | Cell Signaling Technology | 9145 |
| STAT3 | Cell Signaling Technology | 12640 |
| phospho-STAT5 | Cell Signaling Technology | 9359 |
| STAT5 | Cell Signaling Technology | 9363 |
| GAPDH | Cell Signaling Technology | 2118 |
| **Antibodies for flow cytometry:** | | |
| CD45-Percp.Cy5 | BioLegend | 103132 |
| CD11c-BV711 | BioLegend | 117349 |
| CD3-Spark Blue 550 | BioLegend | 100260 |
| CD4-FITC | BioLegend | 100406 |
| CD8-BV510 | BioLegend | 100751 |
| CD80-Spark NIR 685 | BioLegend | 104761 |
| CD206-PE-Dazzle 594 | BioLegend | 141731 |
| CD11b-APC/Fire™ 750 | BioLegend | 101262 |
| PD-1-BUV 805 | BD Sciences | 568609 |
| CTLA-4-BV421 | BioLegend | 369606 |
| Viability 7-AAD | BioLegend | 420403 |

**Supplementary Table 2. Hematological variables for Fn14-WT and Fn14-KO mice.**

|  | **WBC**  **(X 10^3^/µl)** | **Neutrophils**  **(%)** | **Lymphocytes**  **(%)** | **Monocytes**  **(%)** | **Eosinophils**  **(%)** |
| --- | --- | --- | --- | --- | --- |
| **Fn14-WT** | 5.96 +/1.3 | 9.2 +/- 1.5 | 77+/- 2.8 | 2.0+/- 0.3 | 1.4 +/-0.1 |
| **Fn14-KO** | 5.12 +/-2.2 | 8.88+/-2.4 | 83+/-3.2 | 2.5+/-1.0 | 2.5+/-1.8 |

Mean values and standard error of the mean (SEM) for white blood cell count (WBC) and percentage of neutrophils, lymphocytes, monocytes and eosinophils. No statistically significant difference was observed for any of the parameters (ANOVA) between the Fn14-WT and Fn14-KO mice. N=4 per group.

**Supplementary References:**

1. Schneider CA, Rasband WS, Eliceiri KW. NIH Image to ImageJ: 25 years of image analysis. *Nat Methods*. 2012;9(7):671-675. doi:10.1038/nmeth.2089

2. Darmanis S, Sloan SA, Croote D, et al. Single-Cell RNA-Seq Analysis of Infiltrating Neoplastic Cells at the Migrating Front of Human Glioblastoma. *Cell Rep*. 2017;21(5):1399-1410. doi:10.1016/j.celrep.2017.10.030

3. Gao C, Gohel CA, Leng Y, et al. Molecular and spatial profiling of the paraventricular nucleus of the thalamus. *Elife*. 2023;12. doi:10.7554/elife.81818

4. Hao Y, Hao S, Andersen-Nissen E, et al. Integrated analysis of multimodal single-cell data. *Cell*. 2021;184(13):3573-3587.e29. doi:10.1016/j.cell.2021.04.048

5. Hu LS, D’Angelo F, Weiskittel TM, et al. Integrated molecular and multiparametric MRI mapping of high-grade glioma identifies regional biologic signatures. *Nat Commun*. 2023;14(1):6066. doi:10.1038/s41467-023-41559-1

6. Love MI, Huber W, Anders S. Moderated estimation of fold change and dispersion for RNA-seq data with DESeq2. *Genome Biol*. 2014;15(12):550. doi:10.1186/s13059-014-0550-8

7. Xu S, Hu E, Cai Y, et al. Using clusterProfiler to characterize multiomics data. *Nat Protoc*. 2024;19(11):3292-3320. doi:10.1038/s41596-024-01020-z

8. Liberzon A, Subramanian A, Pinchback R, Thorvaldsdóttir H, Tamayo P, Mesirov JP. Molecular signatures database (MSigDB) 3.0. *Bioinformatics*. 2011;27(12):1739-1740. doi:10.1093/bioinformatics/btr260

9. Subramanian A, Tamayo P, Mootha VK, et al. Gene set enrichment analysis: A knowledge-based approach for interpreting genome-wide expression profiles. *Proceedings of the National Academy of Sciences*. 2005;102(43):15545-15550. doi:10.1073/pnas.0506580102

10. Dolgalev I. msigdbr: MSigDB Gene Sets for Multiple Organisms in a Tidy Data Format. Published online 2025.

11. Varn FS, Johnson KC, Martinek J, et al. Glioma progression is shaped by genetic evolution and microenvironment interactions. *Cell*. 2022;185(12):2184-2199.e16. doi:10.1016/j.cell.2022.04.038

12. Leek JT, Johnson WE, Parker HS, Jaffe AE, Storey JD. The <tt>sva</tt> package for removing batch effects and other unwanted variation in high-throughput experiments. *Bioinformatics*. 2012;28(6):882-883. doi:10.1093/bioinformatics/bts034

13. Leek JT, Johnson WE, Parker HS, et al. sva: Surrogate Variable Analysis. . *R package*. 2025;(version 3.56.0). doi:10.18129/B9.bioc.sva

14. Map Entrez Gene identifiers to GenBank Accession Numbers. doi:10.18129/B9.bioc.org.Hs.eg.db

15. Reich M, Liefeld T, Gould J, Lerner J, Tamayo P, Mesirov JP. GenePattern 2.0. *Nat Genet*. 2006;38(5):500-501. doi:10.1038/ng0506-500

16. Sayols S. *Rrvgo: A Bioconductor Package for Interpreting Lists of Gene Ontology Terms*.; 2023. doi:10.17912/micropub.biology.000811

17. Supek F, Bošnjak M, Škunca N, Šmuc T. REVIGO Summarizes and Visualizes Long Lists of Gene Ontology Terms. *PLoS One*. 2011;6(7):e21800. doi:10.1371/journal.pone.0021800

18. Gu Z, Eils R, Schlesner M. Complex heatmaps reveal patterns and correlations in multidimensional genomic data. *Bioinformatics*. 2016;32(18):2847-2849. doi:10.1093/bioinformatics/btw313

19. Gu Z. Complex heatmap visualization. *iMeta*. 2022;1(3):e43. doi:10.1002/imt2.43

20. Wickham H. ggplot2: Elegant Graphics for Data Analysis. Published online 2016.

21. Wickham H. Reshaping Data with the reshape Package. *J Stat Softw*. 2007;21(12):1-20. doi:10.18637/jss.v021.i12

22. Wickham H, Francois R, Henry L, Muller K, Vaughan D. dplyr: A Grammar of Data Manipulation. Published online 2023.

23. Wilke CO. cowplot: Streamlined Plot Theme and Plot Annotations for “ggplot2”. . Published online 2024.

24. Gu Z, Gu L, Eils R, Schlesner M, Brors B. circlize implements and enhances circular visualization in R. *Bioinformatics*. 2014;30(19):2811-2812. doi:10.1093/bioinformatics/btu393

25. McFaline-Figueroa JR, Sun L, Youssef GC, et al. Neoadjuvant anti-PD1 immunotherapy for surgically accessible recurrent glioblastoma: clinical and molecular outcomes of a stage 2 single-arm expansion cohort. *Nat Commun*. 2024;15(1):10757. doi:10.1038/s41467-024-54326-7
